# Immediate and long-term health impact of exposure to gas-mining induced earthquakes and related environmental stressors

**DOI:** 10.1093/eurpub/ckaa244

**Published:** 2021-01-26

**Authors:** Michel L A Dückers, Christos Baliatsas, Peter Spreeuwenberg, Robert A Verheij, Lennart Reifels, C Joris Yzermans

**Affiliations:** 1 Nivel—Netherlands Institute for Health Services Research, Utrecht, The Netherlands; 2 ARQ National Psychotrauma Centre, Diemen, The Netherlands; 3 Faculty of Behavioural and Social Sciences, University of Groningen, Groningen, The Netherlands; 4 Tilburg School of Social and Behavioral Sciences (TRANZO), Tilburg University, Tilburg, The Netherlands; 5 Centre for Mental Health, Melbourne School of Population and Global Health, The University of Melbourne, Carlton, Australia

## Abstract

**Background:**

Little is known about the public health impact of chronic exposure to physical and social stressors in the human environment. Objective of this study was to investigate the immediate and long-term health effects of living in an environment with gas-mining induced earthquakes and related stressors in the Netherlands.

**Methods:**

Data on psychological, somatic and social problems recorded routinely in electronic health records by general practitioners during a 6-year period (2010–2015) were combined with socioeconomic status and seismicity data. To assess immediate health effects of exposure to *M*_L_≥1.5 earthquakes, relative risk ratios were calculated for patients in the week of an earthquake and the week afterwards, and compared to the week before the earthquake. To analyse long-term health effects, relative risks of different groups, adjusted for age, sex and socioeconomic status, were computed per year and compared.

**Results:**

Apart from an increase in suicidality, few immediate health changes were found in an earthquake week or week afterwards. Generally, the prevalence of health problems was higher in the mining province in the first years, but dropped to levels equal to or even below the control group in subsequent years, with lower relative risks observed in more frequently exposed patients.

**Conclusions:**

From a public health perspective, the findings are fascinating. Contrary to our expectation, health problems presented in general practice in the earthquake province decreased during the study period. More frequently exposed populations reported fewer health issues to general practitioners, which might point at health adaptation to chronic exposure to stressors.

## Introduction

In recent decades, the health impact of exposure to disasters has been studied extensively. As a rule, findings point at an increase in physical, psychological and social consequences.^1–6^ The typical pattern in health problems or health care utilization after a disastrous event can be visualized as a sudden peak followed by a gradual decrease over time, with over half of exposed population not developing health problems and a stabilization of population health within 1–2 years.^1^^,^^5^^,^^7^^,^^8^ Disaster health research mostly focused on single events. The few studies considering repeated exposure to natural and human-made disasters suggest that the risk of developing mental health problems across the psychiatric spectrum, including suicidality, is higher in case of repeat exposure.^9^^,^^10^

Despite the growing understanding of the health impact of disasters and relevant risk factors,^1^^,^^4^^,^^8^ little is known about the timing of symptom onset, especially in a context of slow-onset, creeping, recurring or chronic crises that can be accompanied by substantial stressors. Such circumstances are preferably studied with prospective, longitudinal designs that address the prevalence of a broad range of physical, psychological and social health problems, while considering exposure intensity and incorporating control groups. The current study utilizes such a design and examines the development of population health over time in an environment with persistent physical and social risk factors. The objective is to assess the immediate and long-term health effects of living in a region with gas-mining induced earthquakes and related stressors.

### Earthquakes in the Groningen gas field

The population of the Groningen gas field region in the north of the Netherlands has been continually exposed to mining induced earthquakes for over two decades. Gas extraction started in 1962 and seismicity was first recorded in December 1991 when the reservoir reached ∼28% depletion. Currently, the majority of earthquakes in the northern Netherlands is related to gas extraction.^11^ At the end of 2016, a total of 1035 events have been recorded in Groningen, 279 events of *M*_L_≥1.5.^12^ Six earthquakes exceeding M3.0 were recorded. Earthquakes with a magnitude of 4.5–6.4 are considered possible.^12^^,^^13^

In a worldwide review of human-induced earthquakes, the term ‘nuisance earthquakes’ is used for those that cause societal inconvenience; this inconvenience may be physical or psychological, and includes objectionable damage to infrastructure or the environment, public concern, annoyance or distress about ground shaking, noise or environmental effects such as hydrological changes. Nuisance depends on the proximity of people but is difficult to apprehend and context-dependent: ‘Clearly no seismological parameter, e.g. magnitude or intensity of ground shaking, can quantify nuisance because it is dependent on the culture of those affected. Nuisance earthquakes are those that need health-and-safety management.’^11^ The situation in Groningen matches this definition. In the municipalities of Loppersum, Ten Boer and Slochteren, more than 60% of the houses has been damaged, as has over 50% of the houses in the municipalities of Bedum, Eemsmond and Winsum.^14^ Population surveys suggest that the quality of life and trust in the future among inhabitants of the region is negatively affected, and point at an increase in experienced uncertainties, sense of safety, worries, frustration, stress, distrust and a lack of social recognition.^13^^,^^15–17^ These experiences have been attributed to the inability, or even unwillingness, of authorities and the gas company to end or reduce the mining activities and compensate for or repair damaged houses and property.^13^^,^^14^^,^^18–20^

## Methods

### Study design

A longitudinal study was conducted based on routinely recorded electronic health record data from general practices in the earthquake province and in a control group outside this area, considering sex and age, seismic exposure and socioeconomic status.

### Data sources and participants

#### Health records

**Figure 1:**
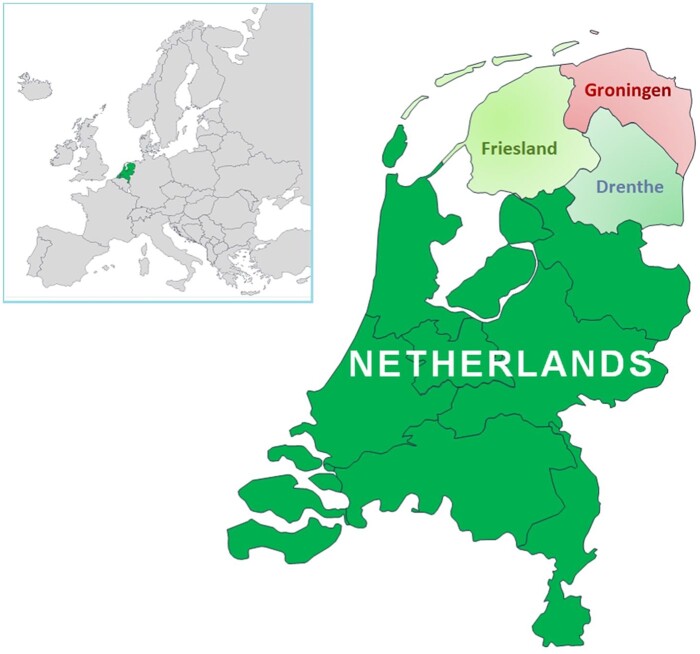
Location of study group (province of Groningen) and control group (provinces of Friesland and Drenthe) in the Netherlands

Health record data were obtained from general practices participating in Nivel Primary Care Database.^21^ The database consists of anonymized data from electronic health records of a representative sample (∼10%) of all general practices in the Netherlands. Every Dutch resident is obliged to be registered at one practice and general practitioners act as gatekeepers to secondary care. Therefore, electronic health records are considered to give a complete picture of population health. The study population included all patients registered in participating practices in the provinces of Groningen, Friesland and Drenthe (figure 1). Health records were available from 16 practices in 2010 (67 629 patients), 27 practices in 2011 (95 716 patients), 52 practices in 2012 (193 685 patients), 56 practices in 2013 (205 668 patients), 62 practices in 2014 (233 121 patients) and 56 practices in 2015 (210 318 patients). Health problems were recorded by general practitioners using the International Classification of Primary Care version 1 (ICPC).^22^ Prevalence estimates were based on disease episodes; each episode contains all ICPC-coded patient contacts (Nielen *et al*. 2019).^23^ ICPC codes were categorized into chronic (irreversible) disorders, long-lasting (reversible) conditions and acute conditions or symptom diagnoses (Nielen *et al*. 2019).^23^ For instance, symptoms such as headache or nausea are classified as an acute condition, meaning that the episode has an ‘end’ after a certain ‘symptom-free’ period, while a Diabetes Mellitus type 2 episode would remain ‘open’ since this concerns a chronic, irreversible condition. Data quality of routine electronic health records is not self-evident,^24^ therefore, only practices that satisfied quality criteria regarding completeness of ICPC coding were included in the study. Health outcomes were analysed as individual symptoms/conditions as well as clusters of diseases. Anxiety, depression, stress reactions, suicidality (suicide and attempts), social problems, non-specific physical and psychological symptoms, and chronic conditions were considered. Also, changes in symptomatology in ICPC-chapters were tested. Supplementary File 1 provides an overview of included health outcomes.

#### Exposure

Seismic activity data were obtained from the Royal Netherlands Meteorological Institute (KNMI), an independent authority that acts as the Dutch national weather service as well as the national research and information centre for meteorology, climate, air quality and seismology. KNMI deploys The Netherlands Seismic and Acoustic Network to monitor seismic events.^25^ The network constitutes a real-time, continuous recording and processing system of high quality seismic and acoustic data from broad band seismometers, borehole geophone stations, accelerometers and infrasound sensors. It detects induced and tectonic earthquakes as well as acoustic signals. When doing statistical analyses on the seismic catalogue, a cut-off value of *M*_L_ = 1.5 is often used.^11^^,^^26^ This corresponds to the minimum magnitude of an earthquake which the geophone network is able to record without failure, independent of its hypocentre or day and day or night timing (NAM, 2015).^27^ Although earthquakes with magnitudes between 1.0 and 3.0 are generally considered ‘light’ and ‘unproblematic’, Vlek described what makes the situation in Groningen unique and ‘hazardously shallow’^13^^,^^28^: the limited depth of the earthquakes (3000 m), the relatively soft and wet surface soil (clay, peat, sand), and the long repetitiveness of seismic activity all contribute to considerable damage and (computed) safety risks over time in this province with a population of 600 000—‘a tectonically inactive region which never needed to be earthquake resistant’.^13^ In this study, the seismic activity data were used to differentiate between no, single and repeat exposure to noticeable earthquakes (*M*_L_≥1.5) in the postal code area of the population. The recorded earthquakes are here considered a proxy for the intensity of noticeable seismicity in the vicinity of the population, including the local impact on livelihood and stress attributed to nuisance earthquakes, housing damage and the governmental response. The analysis has assumed that people living in areas with a higher earthquake frequency and intensity are more likely to be confronted with the impact and uncertainties concerning themselves and their families, neighbourhood or community, even when they did not experience particular earthquakes personally.

#### Socioeconomic status

Neighbourhood socioeconomic status data for the year 2014—which given its stability is a reliable proxy for the period 2010–2015—were retrieved from the Netherlands Institute for Social Research (SCP). The SCP provides ‘status scores’ at a four-digit postal code level for specific years. Socioeconomic status scores are calculated using different survey data sources capturing the average household income, proportion of low family incomes, percentage of low-educated residents and unemployment rates among residents.^29^^,^^30^ These characteristics are combined into a composite score.

### Analysis

Firstly, to assess the immediate health effects of exposure to *M*_L_≥1.5 earthquakes, relative risk (RR) ratios were calculated for patients in the week of an earthquake and the week afterwards, and compared to the week before the earthquake. The count number of patients in a postal code area with particular health problems in a week (before, during, after) was the dependent variable. The multilevel structure of the data (weeks within postal codes areas) was taken into account. Exposure was measured at the postal code level. Cases with repeated exposure, within three weeks of each other, were excluded.

Secondly, to analyse the long-term health effects of exposure to noticeable earthquakes and to living in the earthquake region, the RRs of different groups, adjusted for age, sex and socioeconomic status, were computed per year and compared. Patients in Groningen were divided in three groups: not, once or repeatedly exposed. The exposure groups were compared to the surrounding provinces of Friesland and Drenthe (figure 1). The cross-classified data (individual patients nested in general practitioners and in postal codes) and the count nature of the dependent variable (yearly count of contacts with a specific health problem per patient) were addressed in the statistical models.

All analyses were conducted using a multilevel Poisson regression model using MLwiN (version 2.30). The estimation was done with Restricted Iterative Generalized Least Squares using Penalized Quasi Likelihood first order (first analysis) or second order (second analysis) and allowing for extra-Poisson variation.

## Results

### Seismicity

**Figure 2:**
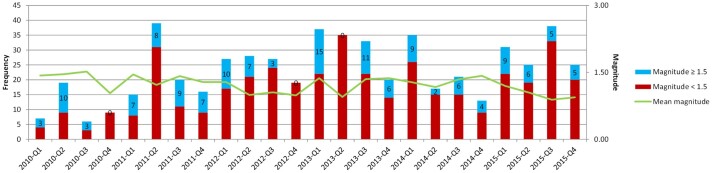
Number of gas-induced earthquakes and mean magnitude per quarter: 2010–2015 (Data Source: Royal Netherlands Meteorological Institute)

Between 2010 and the end of 2015, a total of 562 gas-induced earthquakes were recorded in the study area: 531 in Groningen (94.48%), 28 in Drenthe (4.98%) and 3 in Friesland (0.53%). The number of *M*_L_≥1.5 earthquakes was 145 (24.80%; 135 in Groningen, 9 in Drenthe and 1 in Friesland). All six *M*_L_≥3.0 earthquakes occurred in Groningen. Figure 2 shows the number and magnitude. The frequency is higher in the period 2013–2015 (330) compared to 2010–2012 (232). The mean magnitude is similar in both periods: 1.19 compared to 1.26, respectively, (*P *>* *0.05). The number of *M*_L_≥1.5 earthquakes is 78 and 67, respectively. February 2013 was the month with the most *M*_L_≥1.5 earthquakes.

### Immediate health effects

Single exposure to a noticeable earthquake was not accompanied by an increased risk of reported anxiety, depression, stress reactions, social problems, non-specific symptoms or chronic conditions in the week of the earthquake and the week afterwards. However, as visible in table 1 the risk of suicidality was elevated in the week of the exposure (*RR* = 1.7; CI99 1.04–2.88; *P* < 0.01). Noticeable single exposure also increased symptomatology in the ICPC-chapters Eye (F; *P* < 0.05), Endocrine, Metabolic and Nutritional (T; *P* < 0.05) and Urology (U; *P* < 0.01) in the week of an earthquake. Moreover, the analysis points at an increase of symptoms like nausea, migraine, skin problems and chest pain in the week of a noticeable earthquake (*P* < 0.05). This also applies to neck and shoulder complaints in an earthquake week and dyspnoea, and chest pain in the week after (*P* < 0.05) as well as nausea, musculoskeletal symptoms (i.e. in the back) and skin problems (*P* < 0.01).

**Table 1:** Relative risk of immediate health effects of exposure to noticeable earthquakes (*M*_L_≥1.5)

	Week of earthquake compared to week before			Week after earthquake compared to week before		
	RR	CI99-LO	CI99-HI	RR	CI99-LO	CI99-HI
Anxiety	1.01	0.87	1.16	0.99	0.86	1.15
Depression	1.02	0.90	1.17	0.96	0.84	1.09
Stress reactions	1.01	0.80	1.28	1.00	0.79	1.27
Suicidality	1.73^*^	1.04	2.88	1.46	0.86	2.46
Social problems	0.78	0.51	1.20	1.05	0.71	1.56
Non-specific symptoms	1.03	0.93	1.15	1.01	0.91	1.13
Chronic conditions	1.02	0.92	1.14	0.99	0.89	1.11

*Note.* Relative Risk (RR) ratios were adjusted for age and sex. The number of patients in a postal code area with particular health problems was the dependent variable. The multilevel structure of the data (patients nested in practices) was taken into account. Exposure was measured at the postal code level. Cases with repeat exposure were excluded from the analysis.

* *P *<* *0.01.

### Long-term health effects

**Figure 3:**
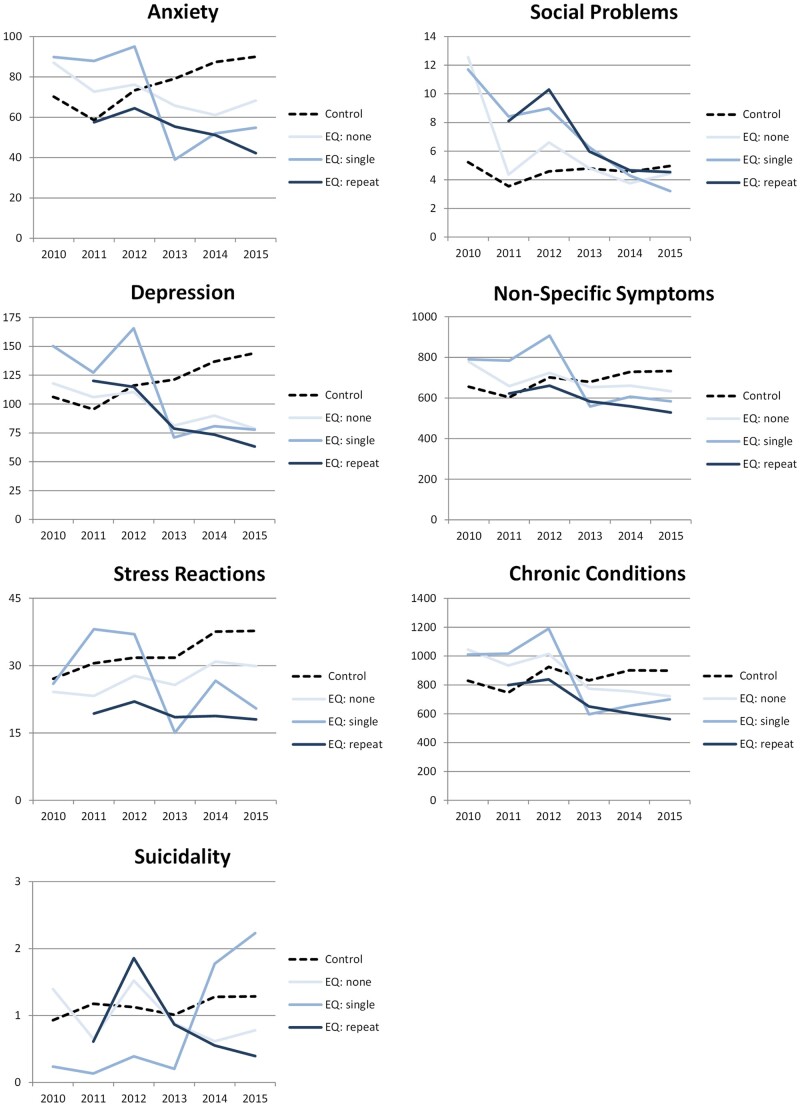
Prevalence of health problems in the earthquake and control regions: 2010–2015 (per 1000 patients) *Note*. Shown here is the prevalence of anxiety, stress reactions, depression, suicidality, social problems, non-specific symptoms and chronic conditions in the study regions between 2010 and 2015. The prevalence in the earthquake (EQ) region is shown in solid lines with darker colours for the more frequently EQ exposed groups and a dotted line for the trend in the control region. Contrary to the control region, most of the trend lines in the earthquake region follow a negative slope. A notable exception is suicidality.

Figure 3 gives an overview of the prevalence of anxiety, depression, stress reactions, suicidality, social problems, non-specific symptoms, and chronic conditions in the earthquake province and the control regions between 2010 and 2015 (detailed information on differences between groups and years for these and other health problems can be found in Supplementary File 2). The exposure groups in Groningen are visualized in solid lines, the control region in a dotted line. More frequently exposed groups are shown in darker solid lines. Unlike the control region, most of the health problems in Groningen follow a negative slope when the timeline evolves. The risk of suicidality, however, ‘increases’ gradually in the single exposure group.

While the prevalence of anxiety increased in the control regions, the slopes in the mining province are all negative. In 2010, the relative risk of the non-exposed and once exposed group is slightly higher (*RR* = 1.24; CI95 0.99–1.55; *P* = NS, respectively, *RR* = 1.28; CI95 0.97–1.68; *P* = NS). Six years later the relative risks of anxiety in Groningen are lower than in the control regions. The relative risk decreased when the exposure to noticeable earthquakes increased, from no exposure (*RR* = 0.76; CI95 0.60–0.96; *P* < 0.02), to single exposure (*RR* = 0.61; CI95 0.45–0.82; *P* < 0.01), to repeat exposure (*RR* = 0.47; CI95 0.35–0.63; *P* < 0.01).

The pattern in depression is similar to anxiety. In 2010, the relative risk in the non-exposed group in Groningen is slightly higher than in the control regions (*RR* = 1.11; CI95 0.91–1.35; *P* = 0.29). The relative risk in the single exposure group is higher (*RR* = 1.42; CI95 1.13–1.78; *P* < 0.01). Six years later the relative risks in the earthquake populations that were not exposed (*RR* = 0.55; CI95 0.44–0.67; *P* < 0.01), once exposed (*RR* = 0.54; CI95 0.40–0.73; *P* < 0.01) and repeatedly exposed (*RR* = 0.44; CI95 0.34–0.56; *P* < 0.01) were lower than in control regions.

In 2010, the relative risk of stress reactions was not larger in the non-exposed (*RR* = 0.89; CI95 0.59–1.36; *P* = NS) and exposed groups (*RR* = 0.96; CI95 0.58–1.59; *P* = NS) compared to the control regions. In 2015, relative risks for non-exposed (*RR* = 0.79; CI95 0.53–1.18; *P* = NS), once exposed (*RR* = 0.54; CI95 0.27–1.09; *P* = NS) and repeatedly exposed (*RR* = 0.48; CI95 0.28–0.82; *P* < 0.01) were lower than in the control region.

Although the general pattern in suicidality looks the same, what is different from the other health problems is that the relative risk of the single exposure group is lower compared to the control regions from 2010 till 2014 (*P* < 0.05 in 2011 and 2013). In 2015, the relative risk of the single exposure group is higher than the control regions. The relative risk of repeatedly exposed patients living in the earthquake region is lower than the single exposure group (*RR* = 0.18; CI95 0.05–0.67; *P* < 0.05).

More social problems were presented to general practitioners in Groningen compared to the control regions in 2010. This applies to the relative risk of the non-exposed (*RR* = 2.40; CI95 1.50–3.84; *P* < 0.01) and single exposure (*RR* = 2.24; CI95 1.28–3.91; *P* < 0.01) groups. Over 6 years the relative risks steadily develop towards the control regions. Eventually, relative risks in none of the exposure groups in Groningen differ from the surrounding regions.

In 2010, the prevalence of non-specific symptoms in the non-exposed (*RR* = 1.19; CI95 1.10–1.28; *P* < 0.01) and single exposure (*RR* = 1.21; CI95 1.10–1.32; *P* < 0.01) population is higher than in the control regions. In 2015, the relative risk of all three exposure groups has dropped below the control regions, again with a reversed association between exposure levels and relative risks: no exposure (*RR* = 0.86; CI95 0.80–0.94; *P* < 0.01), single exposure (*RR* = 0.80; CI95 0.72–0.88; *P* < 0.01) and repeat exposure (*RR* = 0.72; CI95 0.66–0.79; *P* < 0.01). The relative risks vary between the exposure groups (*P* < 0.05).

A comparable development is observed in the prevalence of chronic conditions. In 2010, the relative risk is initially higher in Groningen for the non-exposed (*RR* = 1.26; CI95 1.16–1.37; *P* < 0.01) and once exposed (*RR* = 1.22; CI95 1.13–1.33; *P* < 0.01). Again, after 6 years the relative risk is lower in the three groups with different exposure levels compared to the control regions (*P* < 0.01), with the highest risks in the non-exposed and the single exposure groups (*RR* = 0.80; CI95 0.74–0.87, respectively, *RR* = 0.78; CI95 0.71–0.85; *P* < 0.01). In 2015, the repeat exposure group risk is lower than in the control regions (*RR* = 0.63; CI95 0.57–0.68; *P* < 0.01) and lower than in the non-exposed and once exposed population (*P *< 0.01).

In Supplementary File 3, the quarterly prevalence of health problems in postal codes areas with a *M*_L_ ≥ 3.0 earthquake is visualized. In each postal code, the prevalence decreases over time (like in figure 3), whether the population was confronted with noticeable earthquakes or not. The prevalence of stress reactions, social problems and suicidality was too small to plot.

## Discussion

The analyses shed some light on the immediate and long-term public health effects of living in an environment with noticeable earthquakes and related stressors. When it comes to immediate health impacts linked to noticeable earthquakes, apart from an increase in suicidality, few changes were found in the week of a noticeable earthquake or week afterwards. If a long-term effect was to be expected, as described in the introduction, an increase of health problems in the earthquake population seemed probable, also given the results from earlier surveys and enquiries in the region that were, however, primarily based on self-reported information from a selection of participants, and lacked continuous measurements and a control group. The prevalence of psychological, somatic and social problems (including symptoms) in Groningen was indeed higher from 2010 to 2012, yet since 2013 the prevalence dropped towards (even below) the trend of the control group, with lower risks in more frequently exposed patients.

The study points at public health implications of social and physical environmental circumstances that, despite similarities to disaster settings, do differ from trajectories of post-disaster mental health problems as described in existing epidemiological work which mostly deals with severe, natural and human-made sudden-onset events. With regard to earthquakes in high-income countries, a recent review and meta-analysis of the international literature pointed at, for instance, increased mortality rates from heart attacks and stroke, and increased diabetes in people exposed to earthquakes compared with unexposed people.^6^ This review also found an increase in gastric ulcers and antidepressant and antipsychotic medication use, as well as infectious diseases.^6^ An important difference is that the population in Groningen was not exposed to a heavy earthquake (or other immediate disaster like a flood, hurricane, explosion or severe fire). Thus far, fortunately, no people were killed by collapsing buildings or damage to infrastructure. The study did indicate that exposure to noticeable earthquakes can be accompanied by, mostly transient, stress-related health complaints such as nausea, chest pain and musculoskeletal complaints. The increase in suicidality after single exposure to noticeable earthquakes is not simple to explain. Suicidal behaviour is a very complex, multifactorial problem.

Recent cross-national comparison studies suggest that wealthy and safe countries like the Netherlands are characterized by a higher prevalence of mental health problems like post-traumatic stress disorder, mood, anxiety and substance disorders, and suicide compared to more vulnerable, less affluent countries.^31^^,^^32^ Moreover, the number of psychological and social problems reported to general practitioners in the Netherlands has increased between 2010 and 2014.^33^ This development was also seen in the control regions in this study between 2010 and 2015. However, this trend is abandoned in the earthquake province and moves towards a lower average prevalence of health problems. Perhaps the exposed population is getting used to the changed circumstances of ongoing uncertainty, housing damage and stress, and is adjusting its health profile to a level with less problems. Inhabitants of the mining region might report less health problems to general practitioners over time for several reasons: they experience fewer health problems, believe their problems are not serious enough, expect little benefit from their general practitioner, rely on services from other health care providers, or a combination.

### Strengths and limitations

A major strength of the study is that it utilizes a longitudinal design, combines data on exposure frequency and magnitude with a broad spectrum of health issues, and compares particular risk groups with a control group. Across psychological, physical and social health categories consistent trends were found. At the same time, despite the hazard type being earthquakes, the magnitude of recorded occurrences is substantially lower than in typical disaster research. The results might be indicative for the health impact of exposure to chronic stressors and slow-onset events in the human social and physical environment, and not to ‘real’ disasters. Although some factors could be included or controlled for, a noteworthy limitation is that well-known individual risk factors linked to exposure, damage, social support and lifestyle could not be incorporated; people who experience damage, loss and limited social support are more likely to report poor health.^1^^,^^4^^,^^7^^,^^8^^,^^16^^,^^17^ For these and other reasons, it is important to be careful with assuming causality, especially in relation to suicidality given its low prevalence, complex nature, and the impossibility to differentiate between suicide and attempts in the general practice data.

## Conclusions

Contrary to our expectation, health problems presented in general practice in the earthquake province decreased during the study period. Although it is unclear how the trend will evolve, the patterns across health categories are reassuring because they can be interpreted as health adaptation to chronic exposure to stress. On the other hand, they are disturbing given the increase in suicidality or, alternatively, the potentially decreased tendency to report health issues to general practitioners. The findings might be applicable to other populations confronted with nuisance earthquakes or different types of mild, moderate or even severe stressors attributed to the human physical or social environment. Also, they might give an indication of the long-term health impact of slowly progressing health emergencies like the COVID-19 pandemic in communities across the world.

## Supplementary data

Supplementary data are available at *EURPUB* online.

## Funding

This research received no specific grant from any funding agency, commercial or not-for-profit sectors.

*Conflicts of interest:* None declared.

## Availability of data and materials

The health record data are available from Nivel Primary Care Database (https://www.nivel.nl/en/nivel-primary-care-database) and the work files with computing code are available from the authors upon request.

**Key points**

- Few immediate health effects were found in a population exposed to noticeable earthquakes induced by gas-mining activities in the Netherlands.
- Public health risks were higher in the mining province in the first years but dropped to levels equal to or even below the control group in subsequent years.
- The finding that, over time, lower relative risks were observed in more frequently exposed patients, might point at health adaptation to chronic exposure.
- The findings might be applicable to other populations chronically exposed to mild, moderate or even severe stressors attributed to the physical or social environment of people.
- It is important to continue monitoring the health and wellbeing of the population in the Groningen earthquake region in order to support public health policy.

## Supplementary Material

ckaa244_Supplementary_Data

## References

[1] Norris FH , Friedman MJ, Watson PJ, et al 60,000 disaster victims speak: part I. An empirical review of the empirical literature, 1981-2001. Psychiatry 2002;65:207–39. 12405079 10.1521/psyc.65.3.207.20173

[2] Galea S , Nandi A, Vlahov D. The epidemiology of post-traumatic stress disorder after disasters. Epidemiol Rev 2005;27:78–91. 15958429 10.1093/epirev/mxi003

[3] Herbert R , Moline J, Skloot G, et al The World Trade Center disaster and the health of workers: five-year assessment of a unique medical screening program. Environ Health Perspect 2006;114:1853–8. 17185275 10.1289/ehp.9592 PMC1764159

[4] Yzermans CJ , Van Der Berg B, Dirkzwager AJE. Physical health problems after disasters. In: Neria Y, Galea S, Norris FH, editors, Mental Health and Disasters. New York: Cambridge University Press, 2009: 67–93.

[5] Beaglehole B , Mulder RT, Frampton CM, et al Psychological distress and psychiatric disorder after natural disasters: systematic review and meta-analysis. Br J Psychiatry 2018;213:716–22. 30301477 10.1192/bjp.2018.210

[6] Ripoll Gallardo A , Pacelli B, Alesina M, et al Medium- and long-term health effects of earthquakes in high-income countries: a systematic review and meta-analysis. Int J Epidemiol 2018;47:1317–32. 30053061 10.1093/ije/dyy130

[7] Yzermans CJ , Donker GA, Kerssens JJ, et al Ten Veen PMH. Health problems of victims before and after disaster: a longitudinal study in general practice. Int J Epidemiol 2005;34:820–6. 15860632 10.1093/ije/dyi096

[8] Bonanno GA , Brewin CR, Kaniasty K, La Greca AM. Weighing the costs of disaster: consequences, risks, and resilience in individuals, families, and communities. Psychol Sci Public Interest 2010;11:1–49. 26168411 10.1177/1529100610387086

[9] Reifels L , Mills K, Dückers MLA, O'Donnell ML. Psychiatric epidemiology and disaster exposure in Australia. Epidemiol Psychiatr Sci 2019;28:310–20. 28950925 10.1017/S2045796017000531 PMC7032773

[10] Reifels L , Spittal MJ, Dückers MLA, et al Suicidality risk and (repeat) disaster exposure: findings from a nationally representative population survey. Psychiatry 2018;81:158–72. 30015595 10.1080/00332747.2017.1385049

[11] Foulger GR , Wilson MP, Gluyas JG, et al Global review of human-induced earthquakes. Earth-Sci Rev 2018;178:438–514.

[12] Dost B , Ruigrok E, Spetzler J. Development of seismicity and probabilistic hazard assessment for the Groningen gas field. Neth J Geosci 2017;96:s235–s245.

[13] Vlek C. Rise and reduction of induced earthquakes in the Groningen gas field, 1991–2018. Environ Earth Sci 2019;78:59.

[14] Royal HaskoningDHV. Maatschappelijke effecten inventarisatie van aardbevingen in Noordoost-Groningen 2015, 2016 (Report in Dutch).

[15] Verlinde AA. Veranderingen in kwaliteit van leven Noordoost Groningen 2012-2014 door de gevolgen van gaswinning: Resultaten van een enquête onder stichting WAG deelnemers. Groningen: Stichting WAG, 2014 (Report in Dutch).

[16] Postmes T , Stroebe K, Richardson J, et al Veiligheidsbeleving, gezondheid en toekomstperspectief van Groningers: Wetenschappelijk rapport #2. Groningen: Rijksuniversiteit Groningen, Gronings Perspectief, 2017 (Report in Dutch).

[17] Postmes T , Stroebe K, Richardson J, et al gezondheid en toekomstperspectief van Groningers: Wetenschappelijk rapport #3. Groningen: Rijksuniversiteit Groningen, Gronings Perspectief, 2017 (Report in Dutch).

[18] Ondersteuning Onafhankelijke raadsman. Jaarrapportage: Klachten over de afhandeling van aardbevingsschade in Groningen. Onafhankelijke Raadsman, 2016 (Report in Dutch).

[19] Schmidt A , Boersma K, Groenewegen P. Management strategies in response to an institutional crisis: the case of earthquakes in the Netherlands. Public Adm 2018;1–15.

[20] Van der Voort N , Vanclay F. Social impacts of earthquakes caused by gas extraction in the Province of Groningen, The Netherlands. Environ Impact Assessment Rev 2015;50:1–15.

[21] Nivel Primary Care Database. Utrecht: Nivel. Available at: https://www.nivel.nl/en/nivel-primary-care-database (31 December 2020, date last accessed).

[22] Lamberts H , Wood M. ICPC, International Classification of Primary Care. Oxford: Oxford University Press, 1987.

[23] Nielen MM , Spronk I, Davids R, et al A new method for estimating morbidity rates based on routine electronic medical records in primary care. JMIR Med Inform 2019;7:e11929. 31350839 10.2196/11929 PMC6688441

[24] Verheij RA , Curcin V, Delaney BC, McGilchrist MM. Possible sources of bias in primary care electronic health record data use and reuse. J Med Internet Res 2018;20:e185. 29844010 10.2196/jmir.9134 PMC5997930

[25] Royal Netherlands Meteorological Institute (KNMI). Netherlands Seismic and Acoustic Network. De Bilt: KNMI, 1993.

[26] Hettema MH , Jaarsma B, Schroot BM, Van Yperen GC. An empirical relationship for the seismic activity rate of the Groningen gas field. Neth J Geosci 2017;96:s149–s161.

[27] Nederlands Aardolie Maatschappij (NAM). Hazard and Risk Assessment for Induced Seismicity in Groningen: Interim Update November 2015. Assen: NAM, 2015.

[28] Vlek C. Induced earthquakes from long‐term gas extraction in Groningen, the Netherlands: statistical analysis and prognosis for acceptable‐risk regulation. Risk Analysis 2018;38:1455–73. 29341236 10.1111/risa.12967

[29] Knol F , Veldheer V. Neighbourhood Status Development in The Netherlands 1998-2010. The Hague: Netherlands Institute for Social Research, 2012.

[30] Galobardes B , Shaw M, Lawlor D, et al Indicators of socioeconomic position: methods in social epidemiology. J Epidemiol Community Health 2006;60:47–85. 10.1136/jech.2004.028092 PMC2566160 16415256

[31] Dückers MLA , Alisic E, Brewin CR. A vulnerability paradox in the cross-national prevalence of post-traumatic stress disorder. Br J Psychiatry 2016;209:300–5. 27445357 10.1192/bjp.bp.115.176628

[32] Dückers MLA , Reifels L, De Beurs DP, Brewin CR. The vulnerability paradox in global mental health and its applicability to suicide. Br J Psychiatry 2019;215:588–93. 10.1192/bjp.2019.41 30890196

[33] Magnée T , De Beurs DP, De Bakker DH, Verhaak PF. Consultations in general practices with and without mental health nurses: an observational study from 2010 to 2014. BMJ Open 2016;6:e011579. 10.1136/bmjopen-2016-011579 PMC4964169 27431902

