# Supplementary material for: Immediate and long-term health impact of exposure to gas-mining induced earthquakes and related environmental stressors
Source: Eur J Public Health. 2021 Jan 26;31(4):715–21. doi: 10.1093/eurpub/ckaa244 (PMC8514061; doi:10.1093/eurpub/ckaa244)
Supplement: ckaa244_Supplementary_Data [file ckaa244_supplementary_data.zip › ckaa244-suppl_data/ejph-2020-04-om-0466-File005.pdf]

Supplementary File 1. Overview of included health outcomes

This document gives an overview of the health outcomes used in the study. The coding of the health outcomes in the tables is based on the International Classification of Primary Care (ICPC), version 1 (Lamberts & Wood, 1987). The health outcomes in Table 1 (anxiety, depression, stress reactions, suicidality, social problems, non-specific physical and psychological symptoms, and chronic conditions) were selected based on earlier epidemiological studies of health effects of disasters (e.g. Bonanno et al., 2010; Galea et al., 2005; Norris et al., 2002; Reifels et al., 2017; Reifels et al., 2018; Yzermans et al., 2005; Yzermans et al., 2009; Yzermans et al., 2016). Symptomatology in different ICPC chapters is shown in Table 2. Each code assigned by a general practitioner starts with a capital letter corresponding with its respective ICPC chapter (for examples see Table 2). The numbers 1 to 29 are used to code symptoms and complaints, the numbers 70-99 refer to diagnosed health problems.

Table 1. Health outcomes described in disaster research (symptoms and disorders)

Health outcome	Corresponding ICPC codes
Anxiety	P01 Feeling anxious/nervous/tense<br>P74 Anxiety disorder/anxiety state
Depression	P03 Feeling depressed<br>P76 Depressive disorder
Stress reactions	P02 Acute stress reaction
Suicidality	P77 Suicide/suicide attempt
Social problems	Z01 Poverty/financial problem<br>Z03.01 Inadequate housing<br>Z05.01 Imminent dismissal/resignation from work<br>Z06 Unemployment problem<br>Z12 Relationship problem with partner
Non-specific physical and psychological symptoms	A04 Weakness/tiredness general<br>D01 Abdominal pain/cramps general<br>D02 Abdominal pain epigastric<br>D06 Abdominal pain localized other<br>D09 Nausea<br>D11 Diarrhoea<br>D12 Constipation<br>F01 Eye pain

	F02 Red eye<br>H01 Ear pain/earache<br>H02 Hearing complaint<br>H03 Tinnitus, ringing/buzzing ear<br>H13 Plugged feeling ear<br>K01 Heart pain<br>K02 Pressure/tightness of heart<br>K03 Cardiovascular pain NOS<br>K04 Palpitations/awareness of heart<br>L01 Neck symptom/complain<br>L02 Back symptom/complaint<br>L03 Low back symptom/complaint<br>L04 Chest symptom/complaint<br>L08 Shoulder symptom/complaint<br>L09 Arm symptom/complaint<br>L10 Elbow symptom/complaint<br>L11 Wrist symptom/complaint<br>L12 Hand/finger symptom/complaint<br>L13 Hip symptom/complaint<br>L14 Leg/thigh symptom/complaint<br>L15 Knee symptom/complaint<br>L17 Foot/toe symptom/complaint<br>L18 Muscle pain<br>N01 Headache<br>N02 Tension headache<br>N05 Tingling fingers/feet/toes<br>N17 Vertigo/dizziness<br>P01 Feeling anxious/nervous/tense<br>P02 Acute stress reaction<br>P03 Feeling depressed<br>P04 Feeling/behaving irritable/angry<br>P06 Sleep disturbance<br>P20 Memory disturbance<br>R02 Shortness of breath/dyspnoea<br>R03 Wheezing<br>R04 Breathing problem, other

	R05 Cough<br>R07 Sneezing/nasal congestion<br>R29 Respiratory symptom/complaint, other<br>S01 Pain/tenderness of skin<br>S06 Rash localized<br>S07 Rash generalized<br>T07 Weight gain<br>T08 Weight loss
Chronic Conditions	A12 Allergy/Allergic reaction<br>B80 Iron deficiency-anaemia<br>B81 Pernicious anaemia/folate deficiency<br>B82 Anaemia other/unspecified<br>D89 Inguinal hernia<br>D90 Hiatus hernia<br>D91 Abdominal hernia, other<br>D92 Diverticular disease<br>D93 Spastic colon/Irritable bowel syndrome<br>D94 Chronic enteritis/ulcerative colitis<br>D95 Anal fissure/perianal abscess<br>D96 Hepatomegaly<br>D97 Liver disease NOS<br>D98 Cholecystitis/cholelithiasis<br>F91 Refractive error<br>F92 Cataract<br>F93 Glaucoma<br>F94 Blindness<br>F95 Strabismus<br>H82 Vertiginous syndrome<br>H83 Otosclerosis<br>H84 Presbyacusis<br>H85 Acoustic trauma<br>H86 Deafness<br>K74 Angina pectoris<br>K75 Acute myocardial infarction<br>K76 Ischaemic heart disease, other<br>K77 Heart failure

	K78 Atrial fibrillation/flutter<br>K79 Paroxysmal tachycardia<br>K80 Cardiac arrhythmia NOS<br>K81 Heart/arterial murmur NOS<br>K82 Pulmonary heart disease<br>K83 Heart valve disease NOS<br>K84 Heart disease other<br>K85 Elevated blood pressure<br>K86 Hypertension uncomplicated<br>K87 Hypertension complicated<br>K89 Transient cerebral ischaemia<br>K90 Cerebrovascular accident<br>K91 Atherosclerosis<br>K92 Other peripheral artery disease<br>K93 Pulmonary embolism<br>K94 Phlebitis/thrombophlebitis<br>L83 Neck syndrome<br>L84 Back syndrome w/o radiating pain<br>L85 Acquired deformity of spine<br>L86 Low back pain with radiating pain<br>L88 Rheumatoid arthritis/related condition<br>L89 Osteoarthrosis of hip<br>L90 Osteoarthrosis of knee<br>L91 Osteoarthrosis other<br>L94 Osteochondrosis<br>L95 Osteoporosis<br>L97 Chronic internal trauma of knee<br>L98 Acquired deformity of limb<br>N86 Multiple sclerosis<br>N87 Parkinsonism/Parkinson's disease<br>N88 Epilepsy<br>N89 Migraine<br>P70 Dementia/Alzheimer's disease<br>P71 Organic psychosis, other<br>P72 Schizophrenia<br>P73 Affective psychosis

	P85 Mental retardation<br>P98 Psychosis NOS/other<br>R91 Chronic bronchitis/ bronchiectasis<br>R95 Chronic obstructive pulmonary disease<br>R96 Asthma<br>R97 Allergic rhinitis/Q-fever<br>S86 Dermatitis seborrhoeic<br>S87 Dermatitis/atopic eczema<br>S88 Dermatitis contact/other eczema<br>S91 Psoriasis<br>S97 Chronic ulcer skin<br>S98 Urticaria<br>T81 Goitre<br>T85 Hyperthyroidism/thyrotoxicosis<br>T86 Hypothyroidism/myxoedema<br>T87 Hypoglycaemia<br>T88 Renal glycosuria<br>T90 Diabetes mellitus<br>T93 Lipid disorder

Table 2. Symptomatology in different ICPC chapters

**Chapter**	**Corresponding ICPC codes**
Chapter A. General and unspecified	A01 Pain general/multiple sites<br>A02 Chills<br>A03 Fever<br>A04 Weakness/tiredness general<br>A05 Feeling ill<br>A06 Fainting/syncope<br>A07 Coma<br>A08 Swelling<br>A09 Sweating problem<br>A10 Bleeding/haemorrhage NOS<br>A14 Infantile colic<br>A17 General/unspecified symptoms, infant<br>A29 General symptom/complaint, other
Chapter B. Blood, blood	B02 Lymph gland(s) enlarged/painful

forming organs, lymphatics, spleen	B03 Other symptoms, lymph gland(s)<br>B04 Blood symptom/complaint<br>B29 Symptom/complaint lymph/immune other
Chapter D. Digestive	D01 Abdominal pain/cramps general<br>D02 Abdominal pain epigastric<br>D03 Heartburn<br>D04 Rectal/anal pain<br>D05 Perianal itching<br>D06 Abdominal pain localized, other<br>D08 Flatulence/gas/belching<br>D09 Nausea<br>D10 Vomiting<br>D11 Diarrhoea<br>D12 Constipation<br>D13 Jaundice<br>D14 Haematemesis/vomiting blood<br>D15 Melaena<br>D16 Rectal bleeding<br>D17 Incontinence of bowel<br>D18 Change faeces/bowel movements<br>D19 Teeth/gum symptom/complaint<br>D20 Mouth/tongue/lip symptom/complaint<br>D21 Swallowing problem<br>D22 Worms/other parasites<br>D24 Abdominal mass/swelling<br>D25 Abdominal distension<br>D29 Digestive symptom/complaint other
Chapter F. Eye	F01 Eye pain<br>F02 Red eye<br>F03 Eye discharge<br>F04 Visual floaters/spots<br>F05 Visual disturbance other<br>F13 Eye sensation abnormal<br>F14 Eye movements abnormal<br>F15 Eye appearance abnormal<br>F16 Eyelid symptom/complaint

	F17 Glasses symptom/complaint<br>F18 Contact lens symptom/complaint<br>F29 Eye symptom/complaint other
Chapter H. Ear	H01 Ear pain/earache<br>H02 Hearing complaint<br>H03 Tinnitus, ringing/buzzing ear<br>H04 Ear discharge<br>H05 Bleeding ear<br>H13 Plugged feeling ear<br>H15 Concern with appearance of ears<br>H29 Ear symptom/complaint other<br>H81 Excessive ear wax
Chapter K. Circulatory	K01 Heart pain<br>K02 Pressure/tightness of heart<br>K03 Cardiovascular pain NOS<br>K04 Palpitations/awareness of heart<br>K05 Irregular heartbeat other<br>K06 Prominent veins<br>K07 Swollen ankles/oedema<br>K29 Cardiovascular symptom/complaint, other
Chapter L Musculoskeletal	L01 Neck symptom/complain<br>L02 Back symptom/complaint<br>L03 Low back symptom/complaint<br>L04 Chest symptom/complaint<br>L05 Flank/axilla symptom/complaint<br>L06 Armpit symptom/complaint<br>L07 Jaw symptom/complaint<br>L08 Shoulder symptom/complaint<br>L09 Arm symptom/complaint<br>L10 Elbow symptom/complaint<br>L11 Wrist symptom/complaint<br>L12 Hand/finger symptom/complaint<br>L13 Hip symptom/complaint<br>L14 Leg/thigh symptom/complaint<br>L15 Knee symptom/complaint<br>L16 Ankle symptom/complaint

	L17 Foot/toe symptom/complaint<br>L18 Muscle pain<br>L19 Muscle symptom/complaint NOS<br>L20 Joint symptom/complaint NOS<br>L29 Symptom/complaint musculoskeletal, other/multiple
Chapter N. Neurological	N01 Headache<br>N02 Tension headache<br>N03 Pain face<br>N04 Restless legs<br>N05 Tingling fingers/feet/toes<br>N06 Sensation disturbance other<br>N07 Convulsion/seizure<br>N16 Disturbance of smell/taste<br>N17 Vertigo/dizziness<br>N18 Paralysis/weakness<br>N19 Speech disorder<br>N29 Neurological symptom/complaint, other
Chapter P. Psychological	P01 Feeling anxious/nervous/tense<br>P02 Acute stress reaction<br>P03 Feeling depressed<br>P04 Feeling/behaving irritable/angry<br>P06 Sleep disturbance<br>P20 Memory disturbance
Chapter R. Respiratory	R01 Pain respiratory system<br>R02 Shortness of breath/dyspnoea<br>R03 Wheezing<br>R04 Breathing problem, other<br>R05 Cough<br>R06 Nose bleed/epistaxis<br>R07 Sneezing/nasal congestion<br>R08 Nose symptom/complaint other<br>R09 Sinus symptom/complaint<br>R21 Throat symptom/complaint<br>R22 Symptom/Complaint tonsils<br>R23 Voice symptom/complaint<br>R24 Haemoptysis

	R25 Sputum/phlegm abnormal<br>R29 Respiratory symptom/complaint, other
Chapter S. Skin	S01 Pain/tenderness of skin<br>S02 Pruritus<br>S03 Warts<br>S04 Lump/swelling localized<br>S05 Lumps/swellings generalized<br>S06 Rash localized<br>S07 Rash generalized<br>S08 Skin colour change<br>S20 Corn/callosity<br>S21 Skin texture symptom/complaint<br>S22 Nail symptom/complaint<br>S23 Hair loss/baldness<br>S24 Hair/scalp symptom/complaint<br>S29 Skin symptom/complaint, other
Chapter T. Endocrine, metabolic and nutritional	T01 Excessive thirst<br>T02 Excessive appetite<br>T03 Loss of appetite<br>T04 Feeding problem of infant/child<br>T05 Feeding problem of adult<br>T06 Anorexia nervosa/Bulimia<br>T07 Weight gain<br>T08 Weight loss<br>T10 Growth delay<br>T11 Dehydration<br>T29 Endocrine/met./symptom/complaint, other
Chapter U. Urology	U01 Dysuria/painful urination<br>U02 Urinary frequency/urgency<br>U04 Incontinence urine<br>U05 Urination problems other<br>U06 Haematuria<br>U07 Urine symptom/complaint, other<br>U13 Bladder symptom/complaint, other<br>U14 Kidney symptom/complaint<br>U29 Urinary symptom/complaint, other

Chapter X. Female genital system	X01 Genital pain female<br>X02 Menstrual pain<br>X03 Intermenstrual pain<br>X04 Painful intercourse female<br>X05 Menstruation absent/scanty<br>X06 Menstruation excessive<br>X07 Menstruation irregular/frequent<br>X08 Intermenstrual bleeding<br>X09 Premenstrual symptom/complaint<br>X10 Postponement of menstruation<br>X11 Menopausal symptom/complaint<br>X12 Postmenopausal bleeding<br>X13 Postcoital bleeding<br>X14 Vaginal discharge<br>X15 Vaginal symptom/complaint other<br>X16 Vulval symptom/complaint<br>X17 Pelvis symptom/complaint female<br>X18 Breast pain female<br>X19 Breast lump/mass female<br>X20 Nipple symptom/complaint female<br>X21 Breast symptom/complaint female, other<br>X29 Genital symptom/complaint female, other
Chapter Y. Male genital system	Y01 Pain in penis<br>Y02 Pain in testis/scrotum<br>Y03 Urethral discharge<br>Y04 Penis symptom/complaint other<br>Y05 Scrotum/testis symptom/complaint, other<br>Y06 Prostate symptom/complaint<br>Y07 Impotence symptom/complaint<br>Y08 Sexual function symptom/complaint, other<br>Y16 Breast symptom/complaint male<br>Y29 Genital symptom/complaint male, other
