# Supplementary material for: Immediate and long-term health impact of exposure to gas-mining induced earthquakes and related environmental stressors
Source: Eur J Public Health. 2021 Jan 26;31(4):715–21. doi: 10.1093/eurpub/ckaa244 (PMC8514061; doi:10.1093/eurpub/ckaa244)
Supplement: ckaa244_Supplementary_Data [file ckaa244_supplementary_data.zip › ckaa244-suppl_data/ejph-2020-04-om-0466-File006.pdf]

Supplementary File 2. Long-term health effects

Shown here are the results of the analysis on long-term health effects. The province of Groningen was compared to the control group (provinces of Friesland and Drenthe) while considering earthquake exposure frequency. Relative risk (RR) ratios were adjusted for age and sex. The cross-classified data (patients nested in general practices and in postal codes) was analysed using multilevel statistics, controlling for socioeconomic status at postal code level. Coding is based on Supplementary File 1.

Table 1. Anxiety

Table 2. Depression

Table 3. Stress reactions

Table 4. Suicidality

Table 5. Social problems

Table 6. Non-specific symptoms

Table 7. Chronic conditions

Table 8. ICPC Chapter A. General and unspecified

Table 9. ICPC Chapter B. Blood, blood forming organs, lymphatics, spleen

Table 10. ICPC Chapter D. Digestive

Table 11. ICPC Chapter F. Eye

Table 12. ICPC Chapter H. Ear

Table 13. ICPC Chapter K. Circulatory

Table 14. ICPC Chapter L Musculoskeletal

Table 15. ICPC Chapter N. Neurological

Table 16. ICPC Chapter P. Psychological

Table 17. ICPC Chapter R. Respiratory

Table 18. ICPC Chapter S. Skin

Table 19. ICPC Chapter T. Endocrine, metabolic and nutritional

Table 20. ICPC Chapter U. Urology

Table 21. ICPC Chapter X. Female genital system

Table 22. ICPC Chapter Y. Male genital system

Table 1. Anxiety

RR compared to	Year	Exposure*	RR	CI95-LO	CI95-HI	P
Control group	2010	None	1.2396	0.9894	1.5530	0.0619
Control group	2010	Single	1.2804	0.9744	1.6825	0.0761
Control group	2011	Repeat	1.2404	0.9904	1.5533	0.0606
Control group	2011	Single	1.4997	1.1526	1.9513	0.0025
Control group	2011	Repeat	0.9814	0.7307	1.3182	0.9009
Control group	2012	None	1.0388	0.8296	1.3009	0.7398
Control group	2012	Single	1.2970	0.9860	1.7063	0.0630
Control group	2012	Repeat	0.8787	0.6514	1.1853	0.3971
Control group	2013	None	0.8306	0.6605	1.0444	0.1122
Control group	2013	Single	0.4925	0.3572	0.6790	0.0000
Control group	2013	Repeat	0.6993	0.5185	0.9433	0.0192
Control group	2014	None	0.6989	0.5544	0.8811	0.0024
Control group	2014	Single	0.5950	0.4146	0.8537	0.0048
Control group	2014	Repeat	0.5851	0.4395	0.7788	0.0002
Control group	2015	None	0.7584	0.6003	0.9581	0.0204
Control group	2015	Single	0.6091	0.4507	0.8232	0.0013
Control group	2015	Repeat	0.4693	0.3501	0.6291	0.0000
Groningen: no earthquake $M_L \geq 1.5$	2010	Single	1.0329	0.8408	1.2688	0.7578
Groningen: no earthquake $M_L \geq 1.5$	2011	Single	1.2090	0.9851	1.4838	0.0693
Groningen: no earthquake $M_L \geq 1.5$	2011	Repeat	0.7912	0.6184	1.0124	0.0626
Groningen: no earthquake $M_L \geq 1.5$	2012	Single	1.2485	0.9944	1.5676	0.0559
Groningen: no earthquake $M_L \geq 1.5$	2012	Repeat	0.8458	0.6533	1.0951	0.2039
Groningen: no earthquake $M_L \geq 1.5$	2013	Single	0.5929	0.4456	0.7890	0.0003
Groningen: no earthquake $M_L \geq 1.5$	2013	Repeat	0.8420	0.6468	1.0961	0.2012
Groningen: no earthquake $M_L \geq 1.5$	2014	Single	0.8513	0.6161	1.1763	0.3291
Groningen: no earthquake $M_L \geq 1.5$	2014	Repeat	0.8371	0.6502	1.0778	0.1680
Groningen: no earthquake $M_L \geq 1.5$	2015	Single	0.8032	0.6314	1.0218	0.0743
Groningen: no earthquake $M_L \geq 1.5$	2015	Repeat	0.6188	0.4757	0.8048	0.0003
Groningen: one earthquake $M_L \geq 1.5$	2011	Repeat	0.6544	0.5364	0.7983	0.0000
Groningen: one earthquake $M_L \geq 1.5$	2012	Repeat	0.6775	0.5416	0.8474	0.0006
Groningen: one earthquake $M_L \geq 1.5$	2013	Repeat	1.4201	1.1009	1.8318	0.0069
Groningen: one earthquake $M_L \geq 1.5$	2014	Repeat	0.9834	0.7061	1.3696	0.9210
Groningen: one earthquake $M_L \geq 1.5$	2015	Repeat	0.7704	0.5621	1.0559	0.1048

* Exposure = Number of Earthquakes $M_L \geq 1.5$

Table 2. Depression

RR compared to	Year	Exposure*	RR	CI95-LO	CI95-HI	P
Control group	2010	None	1.1120	0.9132	1.3540	0.2907
Control group	2010	Single	1.4169	1.1294	1.7776	0.0026
Control group	2011	Repeat	1.1121	0.9144	1.3526	0.2873
Control group	2011	Single	1.3343	1.0693	1.6650	0.0107
Control group	2011	Repeat	1.2595	0.9834	1.6130	0.0677
Control group	2012	None	0.9521	0.7872	1.1515	0.6126
Control group	2012	Single	1.4285	1.1460	1.7807	0.0015
Control group	2012	Repeat	0.9908	0.7747	1.2673	0.9414
Control group	2013	None	0.6709	0.5479	0.8216	0.0001
Control group	2013	Single	0.5865	0.4578	0.7514	0.0000
Control group	2013	Repeat	0.6500	0.5029	0.8403	0.0010
Control group	2014	None	0.6582	0.5386	0.8043	0.0000
Control group	2014	Single	0.5912	0.4465	0.7829	0.0002
Control group	2014	Repeat	0.5374	0.4220	0.6843	0.0000
Control group	2015	None	0.5457	0.4434	0.6716	0.0000
Control group	2015	Single	0.5396	0.4003	0.7272	0.0001
Control group	2015	Repeat	0.4380	0.3423	0.5606	0.0000
Groningen: no earthquake $M_L \geq 1.5$	2010	Single	1.2742	1.0808	1.5022	0.0039
Groningen: no earthquake $M_L \geq 1.5$	2011	Single	1.1998	1.0091	1.4266	0.0391
Groningen: no earthquake $M_L \geq 1.5$	2011	Repeat	1.1325	0.9210	1.3926	0.2381
Groningen: no earthquake $M_L \geq 1.5$	2012	Single	1.5005	1.2563	1.7922	0.0000
Groningen: no earthquake $M_L \geq 1.5$	2012	Repeat	1.0407	0.8449	1.2818	0.7074
Groningen: no earthquake $M_L \geq 1.5$	2013	Single	0.8742	0.7012	1.0899	0.2320
Groningen: no earthquake $M_L \geq 1.5$	2013	Repeat	0.9689	0.7681	1.2221	0.7897
Groningen: no earthquake $M_L \geq 1.5$	2014	Single	0.8983	0.7011	1.1510	0.3965
Groningen: no earthquake $M_L \geq 1.5$	2014	Repeat	0.8164	0.6574	1.0139	0.0665
Groningen: no earthquake $M_L \geq 1.5$	2015	Single	0.9887	0.7590	1.2880	0.9331
Groningen: no earthquake $M_L \geq 1.5$	2015	Repeat	0.8027	0.6388	1.0086	0.0592
Groningen: one earthquake $M_L \geq 1.5$	2011	Repeat	0.9439	0.7974	1.1174	0.5023
Groningen: one earthquake $M_L \geq 1.5$	2012	Repeat	0.6936	0.5840	0.8238	0.0000
Groningen: one earthquake $M_L \geq 1.5$	2013	Repeat	1.1083	0.9102	1.3495	0.3060
Groningen: one earthquake $M_L \geq 1.5$	2014	Repeat	0.9089	0.7078	1.1671	0.4539
Groningen: one earthquake $M_L \geq 1.5$	2015	Repeat	0.8118	0.5999	1.0985	0.1766

* Exposure = Number of Earthquakes $M_L \geq 1.5$

Table 3. Stress reactions

RR compared to	Year	Exposure*	RR	CI95-LO	CI95-HI	P
Control group	2010	None	0.8919	0.5860	1.3575	0.5934
Control group	2010	Single	0.9595	0.5791	1.5897	0.8724
Control group	2011	Repeat	0.7606	0.4997	1.1579	0.2019
Control group	2011	Single	1.2478	0.7842	1.9855	0.3502
Control group	2011	Repeat	0.6329	0.3499	1.1447	0.1303
Control group	2012	None	0.8724	0.5952	1.2789	0.4843
Control group	2012	Single	1.1645	0.7063	1.9198	0.5506
Control group	2012	Repeat	0.6920	0.3914	1.2234	0.2053
Control group	2013	None	0.8091	0.5468	1.1970	0.2891
Control group	2013	Single	0.4736	0.2507	0.8946	0.0213
Control group	2013	Repeat	0.5826	0.3243	1.0468	0.0707
Control group	2014	None	0.8227	0.5636	1.2007	0.3117
Control group	2014	Single	0.7087	0.4075	1.2326	0.2227
Control group	2014	Repeat	0.5000	0.2924	0.8550	0.0113
Control group	2015	None	0.7915	0.5332	1.1751	0.2462
Control group	2015	Single	0.5418	0.2705	1.0850	0.0837
Control group	2015	Repeat	0.4772	0.2774	0.8210	0.0075
Groningen: no earthquake $M_L \geq 1.5$	2010	Single	1.0775	0.6959	1.6683	0.7379
Groningen: no earthquake $M_L \geq 1.5$	2011	Single	1.6446	1.0761	2.5133	0.0215
Groningen: no earthquake $M_L \geq 1.5$	2011	Repeat	0.8343	0.4746	1.4665	0.5289
Groningen: no earthquake $M_L \geq 1.5$	2012	Single	1.3375	0.8381	2.1343	0.2226
Groningen: no earthquake $M_L \geq 1.5$	2012	Repeat	0.7946	0.4655	1.3564	0.3994
Groningen: no earthquake $M_L \geq 1.5$	2013	Single	0.5864	0.3203	1.0737	0.0837
Groningen: no earthquake $M_L \geq 1.5$	2013	Repeat	0.7218	0.4119	1.2650	0.2548
Groningen: no earthquake $M_L \geq 1.5$	2014	Single	0.8638	0.5198	1.4353	0.5719
Groningen: no earthquake $M_L \geq 1.5$	2014	Repeat	0.6091	0.3665	1.0122	0.0557
Groningen: no earthquake $M_L \geq 1.5$	2015	Single	0.6867	0.3570	1.3210	0.2602
Groningen: no earthquake $M_L \geq 1.5$	2015	Repeat	0.6045	0.3563	1.0256	0.0620
Groningen: one earthquake $M_L \geq 1.5$	2011	Repeat	0.5046	0.3004	0.8476	0.0097
Groningen: one earthquake $M_L \geq 1.5$	2012	Repeat	0.5919	0.3403	1.0294	0.0633
Groningen: one earthquake $M_L \geq 1.5$	2013	Repeat	1.2581	0.6724	2.3537	0.4726
Groningen: one earthquake $M_L \geq 1.5$	2014	Repeat	0.7076	0.3974	1.2601	0.2401
Groningen: one earthquake $M_L \geq 1.5$	2015	Repeat	0.8856	0.4104	1.9110	0.7569

* Exposure = Number of Earthquakes $M_L \geq 1.5$

Table 4. Suicidality

RR compared to	Year	Exposure*	RR	CI95-LO	CI95-HI	P
Control group	2010	None	1.5027	0.6168	3.6613	0.3701
Control group	2010	Single	0.2551	0.0624	1.0428	0.0572
Control group	2011	Repeat	0.5556	0.2097	1.4721	0.2372
Control group	2011	Single	0.1126	0.0207	0.6122	0.0115
Control group	2011	Repeat	0.5191	0.1337	2.0153	0.3435
Control group	2012	None	1.3501	0.6522	2.7947	0.4187
Control group	2012	Single	0.3462	0.1001	1.1974	0.0938
Control group	2012	Repeat	1.6481	0.5390	5.0398	0.3810
Control group	2013	None	0.8729	0.3983	1.9132	0.7342
Control group	2013	Single	0.2000	0.0453	0.8834	0.0337
Control group	2013	Repeat	0.8557	0.2487	2.9438	0.8048
Control group	2014	None	0.4805	0.2152	1.0728	0.0737
Control group	2014	Single	1.3874	0.4506	4.2718	0.5683
Control group	2014	Repeat	0.4313	0.1450	1.2834	0.1307
Control group	2015	None	0.6072	0.2813	1.3107	0.2038
Control group	2015	Single	1.7367	0.6311	4.7792	0.2852
Control group	2015	Repeat	0.3066	0.1007	0.9339	0.0375
Groningen: no earthquake $M_L \geq 1.5$	2010	Single	0.1698	0.0397	0.7255	0.0167
Groningen: no earthquake $M_L \geq 1.5$	2011	Single	0.2026	0.0327	1.2539	0.0860
Groningen: no earthquake $M_L \geq 1.5$	2011	Repeat	0.9343	0.2035	4.2902	0.9304
Groningen: no earthquake $M_L \geq 1.5$	2012	Single	0.2564	0.0714	0.9214	0.0370
Groningen: no earthquake $M_L \geq 1.5$	2012	Repeat	1.2207	0.3775	3.9472	0.7391
Groningen: no earthquake $M_L \geq 1.5$	2013	Single	0.2291	0.0487	1.0791	0.0624
Groningen: no earthquake $M_L \geq 1.5$	2013	Repeat	0.9803	0.2615	3.6755	0.9765
Groningen: no earthquake $M_L \geq 1.5$	2014	Single	2.8869	0.8959	9.3027	0.0757
Groningen: no earthquake $M_L \geq 1.5$	2014	Repeat	0.8976	0.2701	2.9827	0.8601
Groningen: no earthquake $M_L \geq 1.5$	2015	Single	2.8602	1.0511	7.7835	0.0396
Groningen: no earthquake $M_L \geq 1.5$	2015	Repeat	0.5050	0.1529	1.6674	0.2623
Groningen: one earthquake $M_L \geq 1.5$	2011	Repeat	4.6108	0.7615	27.9162	0.0962
Groningen: one earthquake $M_L \geq 1.5$	2012	Repeat	4.7607	1.4496	15.6350	0.0101
Groningen: one earthquake $M_L \geq 1.5$	2013	Repeat	4.2781	0.9194	19.9068	0.0639
Groningen: one earthquake $M_L \geq 1.5$	2014	Repeat	0.3109	0.0779	1.2405	0.0980
Groningen: one earthquake $M_L \geq 1.5$	2015	Repeat	0.1766	0.0468	0.6668	0.0105

* Exposure = Number of Earthquakes $M_L \geq 1.5$

Table 5. Social problems

RR compared to	Year	Exposure*	RR	CI95-LO	CI95-HI	P
Control group	2010	None	2.4026	1.5046	3.8366	0.0002
Control group	2010	Single	2.2370	1.2798	3.9099	0.0047
Control group	2011	Repeat	1.2322	0.7242	2.0966	0.4413
Control group	2011	Single	2.3756	1.3621	4.1432	0.0023
Control group	2011	Repeat	2.2867	1.1351	4.6067	0.0206
Control group	2012	None	1.4402	0.9002	2.3042	0.1281
Control group	2012	Single	1.9597	1.1357	3.3813	0.0156
Control group	2012	Repeat	2.2467	1.1868	4.2532	0.0129
Control group	2013	None	1.0043	0.6120	1.6483	0.9864
Control group	2013	Single	1.3103	0.7400	2.3204	0.3539
Control group	2013	Repeat	1.2483	0.6141	2.5375	0.5399
Control group	2014	None	0.8248	0.4965	1.3701	0.4570
Control group	2014	Single	0.9426	0.4628	1.9198	0.8706
Control group	2014	Repeat	1.0222	0.5752	1.8168	0.9402
Control group	2015	None	0.8916	0.5439	1.4614	0.6489
Control group	2015	Single	0.6464	0.2603	1.6052	0.3471
Control group	2015	Repeat	0.9127	0.5336	1.5612	0.7388
Groningen: no earthquake $M_L \geq 1.5$	2010	Single	0.9311	0.5889	1.4721	0.7599
Groningen: no earthquake $M_L \geq 1.5$	2011	Single	1.9279	1.1114	3.3444	0.0195
Groningen: no earthquake $M_L \geq 1.5$	2011	Repeat	1.8558	0.9206	3.7408	0.0838
Groningen: no earthquake $M_L \geq 1.5$	2012	Single	1.3607	0.8227	2.2505	0.2302
Groningen: no earthquake $M_L \geq 1.5$	2012	Repeat	1.5600	0.8488	2.8670	0.1521
Groningen: no earthquake $M_L \geq 1.5$	2013	Single	1.3047	0.7479	2.2760	0.3488
Groningen: no earthquake $M_L \geq 1.5$	2013	Repeat	1.2430	0.6152	2.5113	0.5444
Groningen: no earthquake $M_L \geq 1.5$	2014	Single	1.1428	0.5736	2.2767	0.7043
Groningen: no earthquake $M_L \geq 1.5$	2014	Repeat	1.2394	0.6939	2.2135	0.4683
Groningen: no earthquake $M_L \geq 1.5$	2015	Single	0.7250	0.3018	1.7420	0.4722
Groningen: no earthquake $M_L \geq 1.5$	2015	Repeat	1.0237	0.6101	1.7176	0.9292
Groningen: one earthquake $M_L \geq 1.5$	2011	Repeat	0.9626	0.5271	1.7578	0.9012
Groningen: one earthquake $M_L \geq 1.5$	2012	Repeat	1.1465	0.6658	1.9742	0.6220
Groningen: one earthquake $M_L \geq 1.5$	2013	Repeat	0.9527	0.4955	1.8318	0.8845
Groningen: one earthquake $M_L \geq 1.5$	2014	Repeat	1.0845	0.5249	2.2409	0.8266
Groningen: one earthquake $M_L \geq 1.5$	2015	Repeat	1.4120	0.5700	3.4978	0.4561

* Exposure = Number of Earthquakes $M_L \geq 1.5$

Table 6. Non-specific symptoms

RR compared to	Year	Exposure*	RR	CI95-LO	CI95-HI	P
Control group	2010	None	1.1872	1.0970	1.2847	0.0000
Control group	2010	Single	1.2053	1.1014	1.3191	0.0000
Control group	2011	Repeat	1.0899	1.0075	1.1791	0.0319
Control group	2011	Single	1.2974	1.1888	1.4160	0.0000
Control group	2011	Repeat	1.0300	0.9373	1.1319	0.5389
Control group	2012	None	1.0314	0.9546	1.1143	0.4339
Control group	2012	Single	1.2921	1.1831	1.4111	0.0000
Control group	2012	Repeat	0.9411	0.8575	1.0329	0.2010
Control group	2013	None	0.9621	0.8899	1.0401	0.3314
Control group	2013	Single	0.8221	0.7503	0.9008	0.0000
Control group	2013	Repeat	0.8582	0.7824	0.9414	0.0012
Control group	2014	None	0.9066	0.8385	0.9803	0.0139
Control group	2014	Single	0.8326	0.7555	0.9175	0.0002
Control group	2014	Repeat	0.7676	0.7025	0.8388	0.0000
Control group	2015	None	0.8647	0.7985	0.9364	0.0003
Control group	2015	Single	0.7964	0.7231	0.8772	0.0000
Control group	2015	Repeat	0.7219	0.6608	0.7887	0.0000
Groningen: no earthquake $M_L≥1.5$	2010	Single	1.0154	0.9563	1.0781	0.6181
Groningen: no earthquake $M_L≥1.5$	2011	Single	1.1904	1.1200	1.2652	0.0000
Groningen: no earthquake $M_L≥1.5$	2011	Repeat	0.9451	0.8803	1.0146	0.1190
Groningen: no earthquake $M_L≥1.5$	2012	Single	1.2528	1.1763	1.3343	0.0000
Groningen: no earthquake $M_L≥1.5$	2012	Repeat	0.9125	0.8510	0.9785	0.0102
Groningen: no earthquake $M_L≥1.5$	2013	Single	0.8545	0.7982	0.9148	0.0000
Groningen: no earthquake $M_L≥1.5$	2013	Repeat	0.8921	0.8317	0.9568	0.0014
Groningen: no earthquake $M_L≥1.5$	2014	Single	0.9183	0.8535	0.9881	0.0226
Groningen: no earthquake $M_L≥1.5$	2014	Repeat	0.8467	0.7928	0.9043	0.0000
Groningen: no earthquake $M_L≥1.5$	2015	Single	0.9210	0.8590	0.9875	0.0208
Groningen: no earthquake $M_L≥1.5$	2015	Repeat	0.8350	0.7808	0.8929	0.0000
Groningen: one earthquake $M_L≥1.5$	2011	Repeat	0.7939	0.7506	0.8397	0.0000
Groningen: one earthquake $M_L≥1.5$	2012	Repeat	0.7284	0.6879	0.7712	0.0000
Groningen: one earthquake $M_L≥1.5$	2013	Repeat	1.0440	0.9849	1.1065	0.1477
Groningen: one earthquake $M_L≥1.5$	2014	Repeat	0.9220	0.8557	0.9935	0.0332
Groningen: one earthquake $M_L≥1.5$	2015	Repeat	0.9066	0.8340	0.9855	0.0212

* Exposure = Number of Earthquakes $M_L≥1.5$

Table 7. Chronic conditions

RR compared to	Year	Exposure*	RR	CI95-LO	CI95-HI	P
Control group	2010	None	1.2616	1.1627	1.3689	0.0000
Control group	2010	Single	1.2199	1.1170	1.3322	0.0000
Control group	2011	Repeat	1.2517	1.1540	1.3578	0.0000
Control group	2011	Single	1.3623	1.2499	1.4849	0.0000
Control group	2011	Repeat	1.0700	0.9773	1.1715	0.1434
Control group	2012	None	1.0969	1.0120	1.1888	0.0243
Control group	2012	Single	1.2860	1.1796	1.4020	0.0000
Control group	2012	Repeat	0.9063	0.8285	0.9914	0.0316
Control group	2013	None	0.9307	0.8580	1.0097	0.0840
Control group	2013	Single	0.7179	0.6565	0.7850	0.0000
Control group	2013	Repeat	0.7825	0.7152	0.8561	0.0000
Control group	2014	None	0.8384	0.7728	0.9096	0.0000
Control group	2014	Single	0.7264	0.6617	0.7975	0.0000
Control group	2014	Repeat	0.6701	0.6137	0.7317	0.0000
Control group	2015	None	0.8024	0.7392	0.8710	0.0000
Control group	2015	Single	0.7786	0.7096	0.8543	0.0000
Control group	2015	Repeat	0.6251	0.5725	0.6826	0.0000
Groningen: no earthquake $M_L \geq 1.5$	2010	Single	0.9669	0.9239	1.0120	0.1480
Groningen: no earthquake $M_L \geq 1.5$	2011	Single	1.0883	1.0395	1.1394	0.0003
Groningen: no earthquake $M_L \geq 1.5$	2011	Repeat	0.8548	0.8096	0.9025	0.0000
Groningen: no earthquake $M_L \geq 1.5$	2012	Single	1.1724	1.1180	1.2294	0.0000
Groningen: no earthquake $M_L \geq 1.5$	2012	Repeat	0.8262	0.7832	0.8716	0.0000
Groningen: no earthquake $M_L \geq 1.5$	2013	Single	0.7713	0.7308	0.8140	0.0000
Groningen: no earthquake $M_L \geq 1.5$	2013	Repeat	0.8407	0.7956	0.8883	0.0000
Groningen: no earthquake $M_L \geq 1.5$	2014	Single	0.8664	0.8181	0.9176	0.0000
Groningen: no earthquake $M_L \geq 1.5$	2014	Repeat	0.7992	0.7584	0.8422	0.0000
Groningen: no earthquake $M_L \geq 1.5$	2015	Single	0.9703	0.9193	1.0241	0.2741
Groningen: no earthquake $M_L \geq 1.5$	2015	Repeat	0.7790	0.7388	0.8215	0.0000
Groningen: one earthquake $M_L \geq 1.5$	2011	Repeat	0.7854	0.7545	0.8176	0.0000
Groningen: one earthquake $M_L \geq 1.5$	2012	Repeat	0.7047	0.6768	0.7339	0.0000
Groningen: one earthquake $M_L \geq 1.5$	2013	Repeat	1.0900	1.0446	1.1374	0.0001
Groningen: one earthquake $M_L \geq 1.5$	2014	Repeat	0.9224	0.8702	0.9778	0.0066
Groningen: one earthquake $M_L \geq 1.5$	2015	Repeat	0.8029	0.7517	0.8575	0.0000

* Exposure = Number of Earthquakes $M_L \geq 1.5$

Table 8. ICPC Chapter A. General and unspecified

RR compared to	Year	Exposure*	RR	CI95-LO	CI95-HI	P
Control group	2010	None	0.9517	0.7917	1.1442	0.5986
Control group	2010	Single	1.0337	0.8310	1.2858	0.7662
Control group	2011	Repeat	0.9895	0.8254	1.1863	0.9095
Control group	2011	Single	1.3404	1.0868	1.6532	0.0062
Control group	2011	Repeat	1.0724	0.8421	1.3657	0.5708
Control group	2012	None	1.0275	0.8646	1.2212	0.7579
Control group	2012	Single	1.4673	1.1919	1.8063	0.0003
Control group	2012	Repeat	0.8436	0.6622	1.0746	0.1684
Control group	2013	None	1.0304	0.8667	1.2249	0.7347
Control group	2013	Single	0.9077	0.7290	1.1303	0.3869
Control group	2013	Repeat	1.0553	0.8398	1.3261	0.6441
Control group	2014	None	0.9409	0.7908	1.1194	0.4917
Control group	2014	Single	0.9822	0.7671	1.2576	0.8866
Control group	2014	Repeat	0.8347	0.6759	1.0309	0.0934
Control group	2015	None	0.7899	0.6599	0.9453	0.0101
Control group	2015	Single	0.7839	0.6081	1.0104	0.0601
Control group	2015	Repeat	0.7233	0.5870	0.8914	0.0024
Groningen: no earthquake $M_L \geq 1.5$	2010	Single	1.0862	0.9068	1.3009	0.3693
Groningen: no earthquake $M_L \geq 1.5$	2011	Single	1.3545	1.1244	1.6318	0.0014
Groningen: no earthquake $M_L \geq 1.5$	2011	Repeat	1.0839	0.8682	1.3531	0.4767
Groningen: no earthquake $M_L \geq 1.5$	2012	Single	1.4280	1.1893	1.7147	0.0001
Groningen: no earthquake $M_L \geq 1.5$	2012	Repeat	0.8211	0.6583	1.0241	0.0803
Groningen: no earthquake $M_L \geq 1.5$	2013	Single	0.8810	0.7236	1.0727	0.2072
Groningen: no earthquake $M_L \geq 1.5$	2013	Repeat	1.0243	0.8325	1.2604	0.8204
Groningen: no earthquake $M_L \geq 1.5$	2014	Single	1.0440	0.8343	1.3063	0.7067
Groningen: no earthquake $M_L \geq 1.5$	2014	Repeat	0.8873	0.7325	1.0748	0.2216
Groningen: no earthquake $M_L \geq 1.5$	2015	Single	0.9924	0.7909	1.2454	0.9477
Groningen: no earthquake $M_L \geq 1.5$	2015	Repeat	0.9159	0.7540	1.1125	0.3759
Groningen: one earthquake $M_L \geq 1.5$	2011	Repeat	0.8004	0.6633	0.9657	0.0201
Groningen: one earthquake $M_L \geq 1.5$	2012	Repeat	0.5750	0.4748	0.6964	0.0000
Groningen: one earthquake $M_L \geq 1.5$	2013	Repeat	1.1628	0.9684	1.3962	0.1062
Groningen: one earthquake $M_L \geq 1.5$	2014	Repeat	0.8500	0.6763	1.0683	0.1634
Groningen: one earthquake $M_L \geq 1.5$	2015	Repeat	0.9230	0.7142	1.1929	0.5404

* Exposure = Number of Earthquakes $M_L \geq 1.5$

Table 9. ICPC Chapter B. Blood, blood forming organs, lymphatics, spleen

RR compared to	Year	Exposure*	RR	CI95-LO	CI95-HI	P
Control group	2010	None	1.0315	0.7030	1.5136	0.8740
Control group	2010	Single	1.0586	0.6693	1.6743	0.8077
Control group	2011	Repeat	0.8215	0.5666	1.1911	0.2995
Control group	2011	Single	0.8859	0.5679	1.3819	0.5932
Control group	2011	Repeat	0.7709	0.4638	1.2815	0.3157
Control group	2012	None	0.8055	0.5767	1.1250	0.2044
Control group	2012	Single	0.9811	0.6419	1.4995	0.9297
Control group	2012	Repeat	0.8701	0.5513	1.3734	0.5502
Control group	2013	None	1.2191	0.8788	1.6913	0.2354
Control group	2013	Single	0.6683	0.4113	1.0861	0.1038
Control group	2013	Repeat	0.6097	0.3692	1.0069	0.0532
Control group	2014	None	0.9001	0.6432	1.2596	0.5392
Control group	2014	Single	1.0453	0.6484	1.6851	0.8557
Control group	2014	Repeat	0.8446	0.5609	1.2719	0.4188
Control group	2015	None	1.2279	0.8853	1.7031	0.2186
Control group	2015	Single	0.5832	0.3045	1.1168	0.1038
Control group	2015	Repeat	0.6454	0.4327	0.9626	0.0318
Groningen: no earthquake $M_L \geq 1.5$	2010	Single	1.0263	0.6624	1.5900	0.9076
Groningen: no earthquake $M_L \geq 1.5$	2011	Single	1.0784	0.6801	1.7098	0.7483
Groningen: no earthquake $M_L \geq 1.5$	2011	Repeat	0.9385	0.5564	1.5828	0.8117
Groningen: no earthquake $M_L \geq 1.5$	2012	Single	1.2180	0.7951	1.8659	0.3647
Groningen: no earthquake $M_L \geq 1.5$	2012	Repeat	1.0803	0.6825	1.7098	0.7417
Groningen: no earthquake $M_L \geq 1.5$	2013	Single	0.5482	0.3382	0.8886	0.0147
Groningen: no earthquake $M_L \geq 1.5$	2013	Repeat	0.5001	0.3035	0.8241	0.0065
Groningen: no earthquake $M_L \geq 1.5$	2014	Single	1.1613	0.7250	1.8602	0.5337
Groningen: no earthquake $M_L \geq 1.5$	2014	Repeat	0.9384	0.6163	1.4289	0.7670
Groningen: no earthquake $M_L \geq 1.5$	2015	Single	0.4749	0.2518	0.8958	0.0215
Groningen: no earthquake $M_L \geq 1.5$	2015	Repeat	0.5256	0.3527	0.7833	0.0016
Groningen: one earthquake $M_L \geq 1.5$	2011	Repeat	0.8703	0.5328	1.4213	0.5787
Groningen: one earthquake $M_L \geq 1.5$	2012	Repeat	0.8869	0.5744	1.3693	0.5880
Groningen: one earthquake $M_L \geq 1.5$	2013	Repeat	0.9122	0.5323	1.5633	0.7382
Groningen: one earthquake $M_L \geq 1.5$	2014	Repeat	0.8080	0.4877	1.3389	0.4081
Groningen: one earthquake $M_L \geq 1.5$	2015	Repeat	1.1067	0.5632	2.1748	0.7686

* Exposure = Number of Earthquakes $M_L \geq 1.5$

Table 10. ICPC Chapter D. Digestive

RR compared to	Year	Exposure*	RR	CI95-LO	CI95-HI	P
Control group	2010	None	1.2995	1.1465	1.4730	0.0000
Control group	2010	Single	1.2251	1.0572	1.4196	0.0069
Control group	2011	Repeat	1.2119	1.0685	1.3746	0.0028
Control group	2011	Single	1.5657	1.3582	1.8050	0.0000
Control group	2011	Repeat	1.0149	0.8658	1.1897	0.8551
Control group	2012	None	1.1230	0.9922	1.2710	0.0664
Control group	2012	Single	1.4974	1.2966	1.7292	0.0000
Control group	2012	Repeat	0.9386	0.8020	1.0984	0.4295
Control group	2013	None	0.9593	0.8463	1.0875	0.5164
Control group	2013	Single	0.6456	0.5520	0.7551	0.0000
Control group	2013	Repeat	0.7650	0.6541	0.8947	0.0008
Control group	2014	None	0.8570	0.7555	0.9722	0.0165
Control group	2014	Single	0.6158	0.5117	0.7410	0.0000
Control group	2014	Repeat	0.6324	0.5450	0.7338	0.0000
Control group	2015	None	0.8177	0.7171	0.9323	0.0026
Control group	2015	Single	0.7616	0.6440	0.9007	0.0015
Control group	2015	Repeat	0.5648	0.4855	0.6569	0.0000
Groningen: no earthquake $M_L \geq 1.5$	2010	Single	0.9427	0.8484	1.0475	0.2727
Groningen: no earthquake $M_L \geq 1.5$	2011	Single	1.2920	1.1595	1.4396	0.0000
Groningen: no earthquake $M_L \geq 1.5$	2011	Repeat	0.8374	0.7353	0.9537	0.0075
Groningen: no earthquake $M_L \geq 1.5$	2012	Single	1.3334	1.1912	1.4926	0.0000
Groningen: no earthquake $M_L \geq 1.5$	2012	Repeat	0.8358	0.7343	0.9512	0.0066
Groningen: no earthquake $M_L \geq 1.5$	2013	Single	0.6730	0.5907	0.7667	0.0000
Groningen: no earthquake $M_L \geq 1.5$	2013	Repeat	0.7974	0.6996	0.9090	0.0007
Groningen: no earthquake $M_L \geq 1.5$	2014	Single	0.7185	0.6126	0.8428	0.0000
Groningen: no earthquake $M_L \geq 1.5$	2014	Repeat	0.7379	0.6523	0.8347	0.0000
Groningen: no earthquake $M_L \geq 1.5$	2015	Single	0.9314	0.8131	1.0670	0.3056
Groningen: no earthquake $M_L \geq 1.5$	2015	Repeat	0.6907	0.6061	0.7872	0.0000
Groningen: one earthquake $M_L \geq 1.5$	2011	Repeat	0.6482	0.5852	0.7181	0.0000
Groningen: one earthquake $M_L \geq 1.5$	2012	Repeat	0.6269	0.5639	0.6968	0.0000
Groningen: one earthquake $M_L \geq 1.5$	2013	Repeat	1.1849	1.0565	1.3291	0.0038
Groningen: one earthquake $M_L \geq 1.5$	2014	Repeat	1.0269	0.8730	1.2079	0.7486
Groningen: one earthquake $M_L \geq 1.5$	2015	Repeat	0.7416	0.6303	0.8725	0.0003

* Exposure = Number of Earthquakes $M_L \geq 1.5$

Table 11. ICPC Chapter F. Eye

RR compared to	Year	Exposure*	RR	CI95-LO	CI95-HI	P
Control group	2010	None	1.0684	0.8906	1.2818	0.4761
Control group	2010	Single	0.9409	0.7533	1.1753	0.5914
Control group	2011	Repeat	1.0441	0.8716	1.2506	0.6395
Control group	2011	Single	1.4355	1.1671	1.7657	0.0006
Control group	2011	Repeat	0.9566	0.7487	1.2222	0.7227
Control group	2012	None	0.9463	0.7994	1.1203	0.5218
Control group	2012	Single	1.2573	1.0213	1.5479	0.0309
Control group	2012	Repeat	0.9158	0.7245	1.1578	0.4623
Control group	2013	None	0.9280	0.7792	1.1052	0.4018
Control group	2013	Single	0.6372	0.4989	0.8137	0.0003
Control group	2013	Repeat	0.8094	0.6340	1.0333	0.0898
Control group	2014	None	0.7830	0.6601	0.9289	0.0050
Control group	2014	Single	0.7728	0.5871	1.0173	0.0661
Control group	2014	Repeat	0.7153	0.5797	0.8827	0.0018
Control group	2015	None	0.7329	0.6134	0.8757	0.0006
Control group	2015	Single	0.7090	0.5310	0.9466	0.0197
Control group	2015	Repeat	0.7653	0.6242	0.9382	0.0101
Groningen: no earthquake $M_L \geq 1.5$	2010	Single	0.8807	0.7293	1.0635	0.1867
Groningen: no earthquake $M_L \geq 1.5$	2011	Single	1.3749	1.1390	1.6597	0.0009
Groningen: no earthquake $M_L \geq 1.5$	2011	Repeat	0.9162	0.7283	1.1526	0.4549
Groningen: no earthquake $M_L \geq 1.5$	2012	Single	1.3286	1.0995	1.6056	0.0033
Groningen: no earthquake $M_L \geq 1.5$	2012	Repeat	0.9678	0.7785	1.2030	0.7680
Groningen: no earthquake $M_L \geq 1.5$	2013	Single	0.6866	0.5429	0.8684	0.0017
Groningen: no earthquake $M_L \geq 1.5$	2013	Repeat	0.8722	0.6903	1.1022	0.2522
Groningen: no earthquake $M_L \geq 1.5$	2014	Single	0.9870	0.7567	1.2874	0.9228
Groningen: no earthquake $M_L \geq 1.5$	2014	Repeat	0.9135	0.7477	1.1161	0.3762
Groningen: no earthquake $M_L \geq 1.5$	2015	Single	0.9673	0.7319	1.2784	0.8152
Groningen: no earthquake $M_L \geq 1.5$	2015	Repeat	1.0441	0.8558	1.2738	0.6706
Groningen: one earthquake $M_L \geq 1.5$	2011	Repeat	0.6664	0.5431	0.8176	0.0001
Groningen: one earthquake $M_L \geq 1.5$	2012	Repeat	0.7284	0.5961	0.8901	0.0020
Groningen: one earthquake $M_L \geq 1.5$	2013	Repeat	1.2703	0.9946	1.6225	0.0553
Groningen: one earthquake $M_L \geq 1.5$	2014	Repeat	0.9256	0.7061	1.2134	0.5758
Groningen: one earthquake $M_L \geq 1.5$	2015	Repeat	1.0794	0.8042	1.4488	0.6109

* Exposure = Number of Earthquakes $M_L \geq 1.5$

Table 12. ICPC Chapter H. Ear

RR compared to	Year	Exposure*	RR	CI95-LO	CI95-HI	P
Control group	2010	None	1.0073	0.8770	1.1568	0.9184
Control group	2010	Single	1.1277	0.9575	1.3283	0.1500
Control group	2011	Repeat	0.9344	0.8180	1.0673	0.3171
Control group	2011	Single	1.1442	0.9752	1.3426	0.0985
Control group	2011	Repeat	0.8535	0.7111	1.0244	0.0889
Control group	2012	None	1.0099	0.8957	1.1386	0.8720
Control group	2012	Single	1.1458	0.9805	1.3391	0.0869
Control group	2012	Repeat	0.8383	0.7051	0.9966	0.0457
Control group	2013	None	1.0194	0.9064	1.1465	0.7488
Control group	2013	Single	0.8813	0.7511	1.0342	0.1216
Control group	2013	Repeat	0.8143	0.6884	0.9631	0.0165
Control group	2014	None	0.9910	0.8824	1.1128	0.8780
Control group	2014	Single	0.8064	0.6692	0.9716	0.0237
Control group	2014	Repeat	0.7602	0.6540	0.8838	0.0004
Control group	2015	None	0.9002	0.7990	1.0142	0.0839
Control group	2015	Single	0.8736	0.7248	1.0529	0.1559
Control group	2015	Repeat	0.8456	0.7325	0.9762	0.0221
Groningen: no earthquake $M_L \geq 1.5$	2010	Single	1.1196	0.9689	1.2937	0.1256
Groningen: no earthquake $M_L \geq 1.5$	2011	Single	1.2246	1.0524	1.4251	0.0088
Groningen: no earthquake $M_L \geq 1.5$	2011	Repeat	0.9134	0.7666	1.0884	0.3114
Groningen: no earthquake $M_L \geq 1.5$	2012	Single	1.1346	0.9837	1.3086	0.0829
Groningen: no earthquake $M_L \geq 1.5$	2012	Repeat	0.8301	0.7067	0.9750	0.0233
Groningen: no earthquake $M_L \geq 1.5$	2013	Single	0.8646	0.7463	1.0016	0.0525
Groningen: no earthquake $M_L \geq 1.5$	2013	Repeat	0.7988	0.6841	0.9327	0.0045
Groningen: no earthquake $M_L \geq 1.5$	2014	Single	0.8137	0.6831	0.9693	0.0209
Groningen: no earthquake $M_L \geq 1.5$	2014	Repeat	0.7672	0.6681	0.8809	0.0002
Groningen: no earthquake $M_L \geq 1.5$	2015	Single	0.9704	0.8154	1.1549	0.7351
Groningen: no earthquake $M_L \geq 1.5$	2015	Repeat	0.9394	0.8225	1.0729	0.3564
Groningen: one earthquake $M_L \geq 1.5$	2011	Repeat	0.7459	0.6337	0.8780	0.0004
Groningen: one earthquake $M_L \geq 1.5$	2012	Repeat	0.7316	0.6274	0.8532	0.0001
Groningen: one earthquake $M_L \geq 1.5$	2013	Repeat	0.9239	0.7925	1.0772	0.3123
Groningen: one earthquake $M_L \geq 1.5$	2014	Repeat	0.9428	0.7844	1.1332	0.5302
Groningen: one earthquake $M_L \geq 1.5$	2015	Repeat	0.9680	0.8012	1.1696	0.7362

* Exposure = Number of Earthquakes $M_L \geq 1.5$

Table 13. ICPC Chapter K. Circulatory

RR compared to	Year	Exposure*	RR	CI95-LO	CI95-HI	P
Control group	2010	None	1.2476	0.9953	1.5639	0.0549
Control group	2010	Single	1.4611	1.1189	1.9081	0.0054
Control group	2011	Repeat	1.0282	0.8172	1.2937	0.8122
Control group	2011	Single	1.2476	0.9603	1.6209	0.0976
Control group	2011	Repeat	1.0684	0.8001	1.4267	0.6537
Control group	2012	None	1.0647	0.8484	1.3362	0.5882
Control group	2012	Single	1.4499	1.1094	1.8948	0.0065
Control group	2012	Repeat	1.0617	0.7972	1.4139	0.6822
Control group	2013	None	1.0336	0.8225	1.2989	0.7765
Control group	2013	Single	0.7386	0.5513	0.9895	0.0423
Control group	2013	Repeat	0.8848	0.6625	1.1816	0.4069
Control group	2014	None	0.8489	0.6707	1.0744	0.1728
Control group	2014	Single	0.8145	0.5680	1.1679	0.2644
Control group	2014	Repeat	0.7564	0.5743	0.9962	0.0469
Control group	2015	None	0.8892	0.6995	1.1302	0.3371
Control group	2015	Single	0.8778	0.6304	1.2223	0.4403
Control group	2015	Repeat	0.6416	0.4861	0.8468	0.0017
Groningen: no earthquake $M_L \geq 1.5$	2010	Single	1.1711	0.9564	1.4340	0.1264
Groningen: no earthquake $M_L \geq 1.5$	2011	Single	1.2133	0.9760	1.5083	0.0816
Groningen: no earthquake $M_L \geq 1.5$	2011	Repeat	1.0391	0.8076	1.3370	0.7654
Groningen: no earthquake $M_L \geq 1.5$	2012	Single	1.3617	1.0816	1.7144	0.0086
Groningen: no earthquake $M_L \geq 1.5$	2012	Repeat	0.9971	0.7746	1.2837	0.9823
Groningen: no earthquake $M_L \geq 1.5$	2013	Single	0.7145	0.5498	0.9285	0.0119
Groningen: no earthquake $M_L \geq 1.5$	2013	Repeat	0.8560	0.6601	1.1100	0.2409
Groningen: no earthquake $M_L \geq 1.5$	2014	Single	0.9595	0.6887	1.3368	0.8068
Groningen: no earthquake $M_L \geq 1.5$	2014	Repeat	0.8911	0.6920	1.1474	0.3713
Groningen: no earthquake $M_L \geq 1.5$	2015	Single	0.9871	0.7356	1.3246	0.9312
Groningen: no earthquake $M_L \geq 1.5$	2015	Repeat	0.7216	0.5575	0.9340	0.0132
Groningen: one earthquake $M_L \geq 1.5$	2011	Repeat	0.8564	0.7030	1.0432	0.1235
Groningen: one earthquake $M_L \geq 1.5$	2012	Repeat	0.7322	0.5954	0.9005	0.0031
Groningen: one earthquake $M_L \geq 1.5$	2013	Repeat	1.1979	0.9513	1.5085	0.1247
Groningen: one earthquake $M_L \geq 1.5$	2014	Repeat	0.9287	0.6621	1.3026	0.6683
Groningen: one earthquake $M_L \geq 1.5$	2015	Repeat	0.7309	0.5214	1.0246	0.0689

* Exposure = Number of Earthquakes $M_L \geq 1.5$

Table 14. ICPC Chapter L Musculoskeletal

RR compared to	Year	Exposure*	RR	CI95-LO	CI95-HI	P
Control group	2010	None	1.2970	1.1792	1.4267	0.0000
Control group	2010	Single	1.2581	1.1227	1.4099	0.0001
Control group	2011	Repeat	1.0679	0.9708	1.1747	0.1770
Control group	2011	Single	1.2024	1.0758	1.3439	0.0012
Control group	2011	Repeat	0.9969	0.8834	1.1250	0.9599
Control group	2012	None	1.0483	0.9558	1.1496	0.3170
Control group	2012	Single	1.2125	1.0843	1.3557	0.0007
Control group	2012	Repeat	0.9483	0.8427	1.0672	0.3785
Control group	2013	None	1.0604	0.9662	1.1637	0.2166
Control group	2013	Single	0.9189	0.8183	1.0320	0.1531
Control group	2013	Repeat	0.9915	0.8825	1.1141	0.8865
Control group	2014	None	0.9943	0.9062	1.0909	0.9035
Control group	2014	Single	0.9336	0.8238	1.0581	0.2820
Control group	2014	Repeat	0.8957	0.8027	0.9995	0.0489
Control group	2015	None	0.9654	0.8773	1.0623	0.4705
Control group	2015	Single	0.9240	0.8168	1.0452	0.2088
Control group	2015	Repeat	0.8847	0.7932	0.9868	0.0279
Groningen: no earthquake $M_L \geq 1.5$	2010	Single	0.9700	0.8915	1.0554	0.4793
Groningen: no earthquake $M_L \geq 1.5$	2011	Single	1.1260	1.0301	1.2308	0.0090
Groningen: no earthquake $M_L \geq 1.5$	2011	Repeat	0.9335	0.8436	1.0331	0.1834
Groningen: no earthquake $M_L \geq 1.5$	2012	Single	1.1567	1.0569	1.2658	0.0016
Groningen: no earthquake $M_L \geq 1.5$	2012	Repeat	0.9047	0.8202	0.9978	0.0451
Groningen: no earthquake $M_L \geq 1.5$	2013	Single	0.8666	0.7870	0.9542	0.0036
Groningen: no earthquake $M_L \geq 1.5$	2013	Repeat	0.9351	0.8484	1.0306	0.1761
Groningen: no earthquake $M_L \geq 1.5$	2014	Single	0.9390	0.8457	1.0426	0.2387
Groningen: no earthquake $M_L \geq 1.5$	2014	Repeat	0.9009	0.8234	0.9856	0.0228
Groningen: no earthquake $M_L \geq 1.5$	2015	Single	0.9571	0.8663	1.0574	0.3889
Groningen: no earthquake $M_L \geq 1.5$	2015	Repeat	0.9165	0.8360	1.0046	0.0626
Groningen: one earthquake $M_L \geq 1.5$	2011	Repeat	0.8291	0.7600	0.9044	0.0000
Groningen: one earthquake $M_L \geq 1.5$	2012	Repeat	0.7821	0.7174	0.8528	0.0000
Groningen: one earthquake $M_L \geq 1.5$	2013	Repeat	1.0790	0.9887	1.1775	0.0880
Groningen: one earthquake $M_L \geq 1.5$	2014	Repeat	0.9594	0.8620	1.0678	0.4481
Groningen: one earthquake $M_L \geq 1.5$	2015	Repeat	0.9575	0.8523	1.0757	0.4647

* Exposure = Number of Earthquakes $M_L \geq 1.5$

Table 15. ICPC Chapter N. Neurological

RR compared to	Year	Exposure*	RR	CI95-LO	CI95-HI	P
Control group	2010	None	1.2001	0.9935	1.4497	0.0585
Control group	2010	Single	1.4840	1.1876	1.8543	0.0005
Control group	2011	Repeat	1.0543	0.8753	1.2699	0.5777
Control group	2011	Single	1.2588	1.0022	1.5810	0.0478
Control group	2011	Repeat	1.1393	0.8979	1.4455	0.2831
Control group	2012	None	1.0649	0.8955	1.2663	0.4768
Control group	2012	Single	1.2439	0.9918	1.5601	0.0589
Control group	2012	Repeat	0.9723	0.7685	1.2303	0.8153
Control group	2013	None	1.0298	0.8632	1.2286	0.7444
Control group	2013	Single	0.8738	0.6856	1.1136	0.2756
Control group	2013	Repeat	0.8910	0.7042	1.1274	0.3366
Control group	2014	None	0.8315	0.6932	0.9973	0.0467
Control group	2014	Single	0.9611	0.7311	1.2633	0.7759
Control group	2014	Repeat	0.8078	0.6474	1.0079	0.0588
Control group	2015	None	0.8065	0.6677	0.9741	0.0256
Control group	2015	Single	0.8515	0.6546	1.1076	0.2307
Control group	2015	Repeat	0.8365	0.6723	1.0409	0.1094
Groningen: no earthquake $M_L \geq 1.5$	2010	Single	1.2365	1.0237	1.4937	0.0276
Groningen: no earthquake $M_L \geq 1.5$	2011	Single	1.1940	0.9692	1.4709	0.0957
Groningen: no earthquake $M_L \geq 1.5$	2011	Repeat	1.0806	0.8680	1.3454	0.4880
Groningen: no earthquake $M_L \geq 1.5$	2012	Single	1.1681	0.9508	1.4350	0.1390
Groningen: no earthquake $M_L \geq 1.5$	2012	Repeat	0.9131	0.7374	1.1307	0.4045
Groningen: no earthquake $M_L \geq 1.5$	2013	Single	0.8485	0.6773	1.0629	0.1530
Groningen: no earthquake $M_L \geq 1.5$	2013	Repeat	0.8653	0.6963	1.0752	0.1917
Groningen: no earthquake $M_L \geq 1.5$	2014	Single	1.1559	0.8934	1.4956	0.2704
Groningen: no earthquake $M_L \geq 1.5$	2014	Repeat	0.9715	0.7879	1.1980	0.7871
Groningen: no earthquake $M_L \geq 1.5$	2015	Single	1.0558	0.8294	1.3438	0.6593
Groningen: no earthquake $M_L \geq 1.5$	2015	Repeat	1.0373	0.8388	1.2827	0.7355
Groningen: one earthquake $M_L \geq 1.5$	2011	Repeat	0.9051	0.7331	1.1174	0.3536
Groningen: one earthquake $M_L \geq 1.5$	2012	Repeat	0.7817	0.6313	0.9680	0.0240
Groningen: one earthquake $M_L \geq 1.5$	2013	Repeat	1.0198	0.8203	1.2678	0.8601
Groningen: one earthquake $M_L \geq 1.5$	2014	Repeat	0.8405	0.6473	1.0913	0.1922
Groningen: one earthquake $M_L \geq 1.5$	2015	Repeat	0.9825	0.7485	1.2896	0.8988

* Exposure = Number of Earthquakes $M_L \geq 1.5$

Table 16. ICPC Chapter P. Psychological

RR compared to	Year	Exposure*	RR	CI95-LO	CI95-HI	P
Control group	2010	None	1.2399	1.0548	1.4574	0.0091
Control group	2010	Single	1.2921	1.0668	1.5650	0.0088
Control group	2011	Repeat	1.2330	1.0505	1.4471	0.0104
Control group	2011	Single	1.4386	1.1959	1.7307	0.0001
Control group	2011	Repeat	1.0460	0.8526	1.2834	0.6662
Control group	2012	None	1.0553	0.8988	1.2389	0.5111
Control group	2012	Single	1.4321	1.1851	1.7306	0.0002
Control group	2012	Repeat	0.9465	0.7712	1.1618	0.5992
Control group	2013	None	0.7608	0.6455	0.8967	0.0011
Control group	2013	Single	0.4838	0.3892	0.6013	0.0000
Control group	2013	Repeat	0.6269	0.5084	0.7729	0.0000
Control group	2014	None	0.6926	0.5864	0.8180	0.0000
Control group	2014	Single	0.5107	0.3938	0.6624	0.0000
Control group	2014	Repeat	0.5203	0.4253	0.6365	0.0000
Control group	2015	None	0.7121	0.6002	0.8450	0.0001
Control group	2015	Single	0.5257	0.4177	0.6617	0.0000
Control group	2015	Repeat	0.4317	0.3514	0.5305	0.0000
Groningen: no earthquake $M_L \geq 1.5$	2010	Single	1.0422	0.9077	1.1965	0.5578
Groningen: no earthquake $M_L \geq 1.5$	2011	Single	1.1669	1.0165	1.3394	0.0283
Groningen: no earthquake $M_L \geq 1.5$	2011	Repeat	0.8484	0.7197	1.0001	0.0501
Groningen: no earthquake $M_L \geq 1.5$	2012	Single	1.3571	1.1680	1.5770	0.0001
Groningen: no earthquake $M_L \geq 1.5$	2012	Repeat	0.8970	0.7574	1.0623	0.2077
Groningen: no earthquake $M_L \geq 1.5$	2013	Single	0.6359	0.5267	0.7677	0.0000
Groningen: no earthquake $M_L \geq 1.5$	2013	Repeat	0.8240	0.6881	0.9867	0.0352
Groningen: no earthquake $M_L \geq 1.5$	2014	Single	0.7374	0.5849	0.9297	0.0100
Groningen: no earthquake $M_L \geq 1.5$	2014	Repeat	0.7512	0.6311	0.8942	0.0013
Groningen: no earthquake $M_L \geq 1.5$	2015	Single	0.7382	0.6098	0.8938	0.0019
Groningen: no earthquake $M_L \geq 1.5$	2015	Repeat	0.6063	0.5047	0.7283	0.0000
Groningen: one earthquake $M_L \geq 1.5$	2011	Repeat	0.7271	0.6396	0.8265	0.0000
Groningen: one earthquake $M_L \geq 1.5$	2012	Repeat	0.6609	0.5749	0.7597	0.0000
Groningen: one earthquake $M_L \geq 1.5$	2013	Repeat	1.2958	1.0979	1.5293	0.0022
Groningen: one earthquake $M_L \geq 1.5$	2014	Repeat	1.0187	0.8060	1.2875	0.8771
Groningen: one earthquake $M_L \geq 1.5$	2015	Repeat	0.8212	0.6504	1.0370	0.0980

* Exposure = Number of Earthquakes $M_L \geq 1.5$

Table 17. ICPC Chapter R. Respiratory

RR compared to	Year	Exposure*	RR	CI95-LO	CI95-HI	P
Control group	2010	None	1.0516	0.9294	1.1897	0.4247
Control group	2010	Single	1.1747	1.0161	1.3580	0.0295
Control group	2011	Repeat	0.9963	0.8826	1.1247	0.9526
Control group	2011	Single	1.2046	1.0469	1.3861	0.0093
Control group	2011	Repeat	1.0071	0.8590	1.1808	0.9303
Control group	2012	None	0.9379	0.8365	1.0515	0.2717
Control group	2012	Single	1.1799	1.0253	1.3578	0.0209
Control group	2012	Repeat	0.8957	0.7664	1.0468	0.1661
Control group	2013	None	0.9003	0.7997	1.0135	0.0821
Control group	2013	Single	0.9061	0.7821	1.0497	0.1890
Control group	2013	Repeat	0.8697	0.7442	1.0163	0.0790
Control group	2014	None	0.8874	0.7888	0.9984	0.0469
Control group	2014	Single	0.8399	0.7070	0.9978	0.0471
Control group	2014	Repeat	0.8341	0.7244	0.9604	0.0117
Control group	2015	None	0.8302	0.7371	0.9351	0.0022
Control group	2015	Single	0.6853	0.5716	0.8216	0.0000
Control group	2015	Repeat	0.7408	0.6458	0.8498	0.0000
Groningen: no earthquake $M_L \geq 1.5$	2010	Single	1.1171	0.9921	1.2578	0.0674
Groningen: no earthquake $M_L \geq 1.5$	2011	Single	1.2091	1.0689	1.3676	0.0025
Groningen: no earthquake $M_L \geq 1.5$	2011	Repeat	1.0108	0.8750	1.1678	0.8836
Groningen: no earthquake $M_L \geq 1.5$	2012	Single	1.2580	1.1116	1.4238	0.0003
Groningen: no earthquake $M_L \geq 1.5$	2012	Repeat	0.9550	0.8298	1.0991	0.5212
Groningen: no earthquake $M_L \geq 1.5$	2013	Single	1.0065	0.8797	1.1515	0.9249
Groningen: no earthquake $M_L \geq 1.5$	2013	Repeat	0.9660	0.8366	1.1154	0.6375
Groningen: no earthquake $M_L \geq 1.5$	2014	Single	0.9464	0.8066	1.1106	0.4999
Groningen: no earthquake $M_L \geq 1.5$	2014	Repeat	0.9399	0.8254	1.0704	0.3502
Groningen: no earthquake $M_L \geq 1.5$	2015	Single	0.8254	0.6985	0.9753	0.0242
Groningen: no earthquake $M_L \geq 1.5$	2015	Repeat	0.8923	0.7859	1.0130	0.0783
Groningen: one earthquake $M_L \geq 1.5$	2011	Repeat	0.8360	0.7356	0.9502	0.0061
Groningen: one earthquake $M_L \geq 1.5$	2012	Repeat	0.7591	0.6673	0.8636	0.0000
Groningen: one earthquake $M_L \geq 1.5$	2013	Repeat	0.9598	0.8407	1.0957	0.5436
Groningen: one earthquake $M_L \geq 1.5$	2014	Repeat	0.9931	0.8446	1.1678	0.9334
Groningen: one earthquake $M_L \geq 1.5$	2015	Repeat	1.0810	0.9025	1.2948	0.3975

* Exposure = Number of Earthquakes $M_L \geq 1.5$

Table 18. ICPC Chapter S. Skin

RR compared to	Year	Exposure*	RR	CI95-LO	CI95-HI	P
Control group	2010	None	1.2423	1.0690	1.4436	0.0046
Control group	2010	Single	1.2504	1.0434	1.4985	0.0155
Control group	2011	Repeat	1.0592	0.9165	1.2241	0.4360
Control group	2011	Single	1.3506	1.1374	1.6039	0.0006
Control group	2011	Repeat	1.1647	0.9619	1.4102	0.1182
Control group	2012	None	1.0227	0.8909	1.1739	0.7502
Control group	2012	Single	1.2278	1.0284	1.4660	0.0232
Control group	2012	Repeat	1.0833	0.8977	1.3073	0.4043
Control group	2013	None	0.9330	0.8145	1.0688	0.3170
Control group	2013	Single	1.0082	0.8445	1.2037	0.9278
Control group	2013	Repeat	1.0696	0.8929	1.2813	0.4651
Control group	2014	None	0.9124	0.7974	1.0441	0.1827
Control group	2014	Single	0.8819	0.7211	1.0784	0.2208
Control group	2014	Repeat	0.9965	0.8463	1.1733	0.9662
Control group	2015	None	0.8678	0.7530	1.0001	0.0502
Control group	2015	Single	0.7022	0.5575	0.8844	0.0027
Control group	2015	Repeat	0.8846	0.7512	1.0415	0.1411
Groningen: no earthquake $M_L \geq 1.5$	2010	Single	1.0066	0.8625	1.1747	0.9339
Groningen: no earthquake $M_L \geq 1.5$	2011	Single	1.2751	1.0889	1.4933	0.0026
Groningen: no earthquake $M_L \geq 1.5$	2011	Repeat	1.0996	0.9198	1.3146	0.2973
Groningen: no earthquake $M_L \geq 1.5$	2012	Single	1.2006	1.0179	1.4161	0.0300
Groningen: no earthquake $M_L \geq 1.5$	2012	Repeat	1.0593	0.8886	1.2627	0.5208
Groningen: no earthquake $M_L \geq 1.5$	2013	Single	1.0806	0.9160	1.2748	0.3577
Groningen: no earthquake $M_L \geq 1.5$	2013	Repeat	1.1464	0.9692	1.3561	0.1108
Groningen: no earthquake $M_L \geq 1.5$	2014	Single	0.9665	0.8003	1.1672	0.7232
Groningen: no earthquake $M_L \geq 1.5$	2014	Repeat	1.0921	0.9383	1.2711	0.2553
Groningen: no earthquake $M_L \geq 1.5$	2015	Single	0.8091	0.6505	1.0065	0.0572
Groningen: no earthquake $M_L \geq 1.5$	2015	Repeat	1.0193	0.8712	1.1926	0.8114
Groningen: one earthquake $M_L \geq 1.5$	2011	Repeat	0.8624	0.7327	1.0150	0.0749
Groningen: one earthquake $M_L \geq 1.5$	2012	Repeat	0.8823	0.7438	1.0465	0.1505
Groningen: one earthquake $M_L \geq 1.5$	2013	Repeat	1.0609	0.9030	1.2464	0.4722
Groningen: one earthquake $M_L \geq 1.5$	2014	Repeat	1.1299	0.9342	1.3667	0.2080
Groningen: one earthquake $M_L \geq 1.5$	2015	Repeat	1.2597	0.9963	1.5927	0.0537

* Exposure = Number of Earthquakes $M_L \geq 1.5$

Table 19. ICPC Chapter T. Endocrine, metabolic and nutritional

RR compared to	Year	Exposure*	RR	CI95-LO	CI95-HI	P
Control group	2010	None	0.7991	0.5274	1.2108	0.2901
Control group	2010	Single	1.1292	0.7227	1.7644	0.5935
Control group	2011	Repeat	0.8233	0.5508	1.2306	0.3431
Control group	2011	Single	1.2549	0.8125	1.9380	0.3059
Control group	2011	Repeat	0.7530	0.4274	1.3268	0.3263
Control group	2012	None	1.0355	0.7507	1.4284	0.8316
Control group	2012	Single	1.2319	0.7945	1.9102	0.3514
Control group	2012	Repeat	0.8637	0.5220	1.4290	0.5684
Control group	2013	None	0.8810	0.6208	1.2501	0.4779
Control group	2013	Single	0.8572	0.5291	1.3885	0.5312
Control group	2013	Repeat	0.9246	0.5689	1.5026	0.7516
Control group	2014	None	0.8151	0.5773	1.1509	0.2454
Control group	2014	Single	1.0232	0.5970	1.7536	0.9335
Control group	2014	Repeat	0.7991	0.5220	1.2232	0.3018
Control group	2015	None	0.7828	0.5390	1.1369	0.1984
Control group	2015	Single	1.1171	0.6227	2.0038	0.7104
Control group	2015	Repeat	0.5911	0.3659	0.9549	0.0316
Groningen: no earthquake $M_L \geq 1.5$	2010	Single	1.4132	0.8841	2.2588	0.1484
Groningen: no earthquake $M_L \geq 1.5$	2011	Single	1.5242	0.9362	2.4815	0.0901
Groningen: no earthquake $M_L \geq 1.5$	2011	Repeat	0.9147	0.4977	1.6811	0.7740
Groningen: no earthquake $M_L \geq 1.5$	2012	Single	1.1897	0.7530	1.8796	0.4567
Groningen: no earthquake $M_L \geq 1.5$	2012	Repeat	0.8342	0.4987	1.3953	0.4897
Groningen: no earthquake $M_L \geq 1.5$	2013	Single	0.9730	0.5793	1.6344	0.9177
Groningen: no earthquake $M_L \geq 1.5$	2013	Repeat	1.0496	0.6241	1.7652	0.8552
Groningen: no earthquake $M_L \geq 1.5$	2014	Single	1.2554	0.7140	2.2070	0.4295
Groningen: no earthquake $M_L \geq 1.5$	2014	Repeat	0.9804	0.6132	1.5676	0.9343
Groningen: no earthquake $M_L \geq 1.5$	2015	Single	1.4270	0.7751	2.6272	0.2535
Groningen: no earthquake $M_L \geq 1.5$	2015	Repeat	0.7552	0.4414	1.2921	0.3055
Groningen: one earthquake $M_L \geq 1.5$	2011	Repeat	0.6001	0.3353	1.0740	0.0855
Groningen: one earthquake $M_L \geq 1.5$	2012	Repeat	0.7012	0.4037	1.2177	0.2075
Groningen: one earthquake $M_L \geq 1.5$	2013	Repeat	1.0786	0.6216	1.8717	0.7878
Groningen: one earthquake $M_L \geq 1.5$	2014	Repeat	0.7810	0.4424	1.3787	0.3939
Groningen: one earthquake $M_L \geq 1.5$	2015	Repeat	0.5292	0.2680	1.0448	0.0667

* Exposure = Number of Earthquakes $M_L \geq 1.5$

Table 20. ICPC Chapter U. Urology

RR compared to	Year	Exposure*	RR	CI95-LO	CI95-HI	P
Control group	2010	None	1.3240	1.0718	1.6355	0.0092
Control group	2010	Single	1.3386	1.0428	1.7184	0.0221
Control group	2011	Repeat	1.3067	1.0526	1.6222	0.0153
Control group	2011	Single	1.8129	1.4170	2.3195	0.0000
Control group	2011	Repeat	1.4481	1.1137	1.8829	0.0057
Control group	2012	None	1.0695	0.8691	1.3161	0.5254
Control group	2012	Single	1.6637	1.3053	2.1205	0.0000
Control group	2012	Repeat	1.1769	0.9114	1.5199	0.2118
Control group	2013	None	1.0761	0.8785	1.3180	0.4787
Control group	2013	Single	0.8951	0.6943	1.1541	0.3928
Control group	2013	Repeat	1.0667	0.8314	1.3686	0.6115
Control group	2014	None	0.8118	0.6575	1.0023	0.0526
Control group	2014	Single	0.9890	0.7288	1.3420	0.9434
Control group	2014	Repeat	0.8750	0.6872	1.1141	0.2786
Control group	2015	None	0.8718	0.7010	1.0842	0.2174
Control group	2015	Single	0.7103	0.5230	0.9646	0.0285
Control group	2015	Repeat	1.0322	0.8130	1.3105	0.7947
Groningen: no earthquake $M_L \geq 1.5$	2010	Single	1.0110	0.8282	1.2340	0.9145
Groningen: no earthquake $M_L \geq 1.5$	2011	Single	1.3872	1.1216	1.7157	0.0025
Groningen: no earthquake $M_L \geq 1.5$	2011	Repeat	1.1084	0.8791	1.3974	0.3843
Groningen: no earthquake $M_L \geq 1.5$	2012	Single	1.5554	1.2621	1.9169	0.0000
Groningen: no earthquake $M_L \geq 1.5$	2012	Repeat	1.1006	0.8799	1.3765	0.4012
Groningen: no earthquake $M_L \geq 1.5$	2013	Single	0.8318	0.6674	1.0368	0.1014
Groningen: no earthquake $M_L \geq 1.5$	2013	Repeat	0.9914	0.8001	1.2285	0.9372
Groningen: no earthquake $M_L \geq 1.5$	2014	Single	1.2184	0.9226	1.6089	0.1638
Groningen: no earthquake $M_L \geq 1.5$	2014	Repeat	1.0779	0.8689	1.3373	0.4951
Groningen: no earthquake $M_L \geq 1.5$	2015	Single	0.8147	0.6214	1.0681	0.1380
Groningen: no earthquake $M_L \geq 1.5$	2015	Repeat	1.1842	0.9518	1.4734	0.1294
Groningen: one earthquake $M_L \geq 1.5$	2011	Repeat	0.7990	0.6652	0.9596	0.0163
Groningen: one earthquake $M_L \geq 1.5$	2012	Repeat	0.7076	0.5933	0.8438	0.0001
Groningen: one earthquake $M_L \geq 1.5$	2013	Repeat	1.1918	0.9989	1.4221	0.0515
Groningen: one earthquake $M_L \geq 1.5$	2014	Repeat	0.8848	0.6722	1.1646	0.3826
Groningen: one earthquake $M_L \geq 1.5$	2015	Repeat	1.4536	1.0800	1.9564	0.0136

* Exposure = Number of Earthquakes $M_L \geq 1.5$

Table 21. ICPC Chapter X. Female genital system

RR compared to	Year	Exposure*	RR	CI95-LO	CI95-HI	P
Control group	2010	None	1.4100	1.2126	1.6397	0.0000
Control group	2010	Single	1.3639	1.1275	1.6500	0.0014
Control group	2011	Repeat	1.1117	0.9600	1.2874	0.1570
Control group	2011	Single	1.2332	1.0265	1.4815	0.0251
Control group	2011	Repeat	0.9883	0.8001	1.2207	0.9127
Control group	2012	None	1.1818	1.0335	1.3513	0.0146
Control group	2012	Single	1.1734	0.9666	1.4245	0.1061
Control group	2012	Repeat	0.9497	0.7706	1.1705	0.6286
Control group	2013	None	1.2028	1.0468	1.3820	0.0092
Control group	2013	Single	1.2042	0.9946	1.4579	0.0568
Control group	2013	Repeat	0.9536	0.7765	1.1711	0.6505
Control group	2014	None	1.0883	0.9522	1.2439	0.2147
Control group	2014	Single	1.0445	0.8434	1.2935	0.6899
Control group	2014	Repeat	0.8715	0.7309	1.0392	0.1256
Control group	2015	None	1.0460	0.9048	1.2094	0.5430
Control group	2015	Single	1.0851	0.8563	1.3750	0.4989
Control group	2015	Repeat	0.8761	0.7329	1.0473	0.1464
Groningen: no earthquake $M_L \geq 1.5$	2010	Single	0.9673	0.8106	1.1543	0.7125
Groningen: no earthquake $M_L \geq 1.5$	2011	Single	1.1092	0.9248	1.3304	0.2639
Groningen: no earthquake $M_L \geq 1.5$	2011	Repeat	0.8889	0.7210	1.0959	0.2703
Groningen: no earthquake $M_L \geq 1.5$	2012	Single	0.9929	0.8198	1.2027	0.9422
Groningen: no earthquake $M_L \geq 1.5$	2012	Repeat	0.8037	0.6550	0.9862	0.0363
Groningen: no earthquake $M_L \geq 1.5$	2013	Single	1.0012	0.8261	1.2134	0.9905
Groningen: no earthquake $M_L \geq 1.5$	2013	Repeat	0.7928	0.6459	0.9732	0.0264
Groningen: no earthquake $M_L \geq 1.5$	2014	Single	0.9598	0.7768	1.1858	0.7035
Groningen: no earthquake $M_L \geq 1.5$	2014	Repeat	0.8008	0.6720	0.9543	0.0131
Groningen: no earthquake $M_L \geq 1.5$	2015	Single	1.0373	0.8212	1.3104	0.7585
Groningen: no earthquake $M_L \geq 1.5$	2015	Repeat	0.8375	0.6961	1.0077	0.0602
Groningen: one earthquake $M_L \geq 1.5$	2011	Repeat	0.8014	0.6505	0.9873	0.0376
Groningen: one earthquake $M_L \geq 1.5$	2012	Repeat	0.8094	0.6468	1.0129	0.0646
Groningen: one earthquake $M_L \geq 1.5$	2013	Repeat	0.7919	0.6397	0.9804	0.0322
Groningen: one earthquake $M_L \geq 1.5$	2014	Repeat	0.8344	0.6697	1.0397	0.1067
Groningen: one earthquake $M_L \geq 1.5$	2015	Repeat	0.8074	0.6254	1.0423	0.1006

* Exposure = Number of Earthquakes $M_L \geq 1.5$

Table 22. ICPC Chapter Y. Male genital system

RR compared to	Year	Exposure*	RR	CI95-LO	CI95-HI	P
Control group	2010	None	1.6168	1.2337	2.1188	0.0005
Control group	2010	Single	1.2248	0.8952	1.6758	0.2049
Control group	2011	Repeat	1.1311	0.8535	1.4989	0.3913
Control group	2011	Single	1.6673	1.2407	2.2408	0.0007
Control group	2011	Repeat	0.8590	0.6000	1.2298	0.4065
Control group	2012	None	0.9614	0.7379	1.2525	0.7703
Control group	2012	Single	2.1559	1.6251	2.8600	0.0000
Control group	2012	Repeat	0.7711	0.5489	1.0832	0.1338
Control group	2013	None	0.8975	0.6800	1.1845	0.4449
Control group	2013	Single	0.6309	0.4466	0.8913	0.0090
Control group	2013	Repeat	0.8319	0.5930	1.1671	0.2868
Control group	2014	None	0.8131	0.6195	1.0670	0.1357
Control group	2014	Single	0.6074	0.3980	0.9270	0.0208
Control group	2014	Repeat	0.5888	0.4324	0.8018	0.0008
Control group	2015	None	0.7259	0.5419	0.9723	0.0317
Control group	2015	Single	0.5484	0.3331	0.9027	0.0182
Control group	2015	Repeat	0.5493	0.4013	0.7518	0.0002
Groningen: no earthquake $M_L \geq 1.5$	2010	Single	0.7575	0.5967	0.9617	0.0226
Groningen: no earthquake $M_L \geq 1.5$	2011	Single	1.4741	1.1347	1.9151	0.0037
Groningen: no earthquake $M_L \geq 1.5$	2011	Repeat	0.7595	0.5447	1.0590	0.1048
Groningen: no earthquake $M_L \geq 1.5$	2012	Single	2.2425	1.7388	2.8922	0.0000
Groningen: no earthquake $M_L \geq 1.5$	2012	Repeat	0.8021	0.5848	1.1000	0.1711
Groningen: no earthquake $M_L \geq 1.5$	2013	Single	0.7030	0.5032	0.9821	0.0389
Groningen: no earthquake $M_L \geq 1.5$	2013	Repeat	0.9270	0.6684	1.2857	0.6495
Groningen: no earthquake $M_L \geq 1.5$	2014	Single	0.7470	0.4975	1.1218	0.1597
Groningen: no earthquake $M_L \geq 1.5$	2014	Repeat	0.7242	0.5375	0.9758	0.0339
Groningen: no earthquake $M_L \geq 1.5$	2015	Single	0.7555	0.4649	1.2276	0.2576
Groningen: no earthquake $M_L \geq 1.5$	2015	Repeat	0.7568	0.5497	1.0419	0.0876
Groningen: one earthquake $M_L \geq 1.5$	2011	Repeat	0.5152	0.3894	0.6817	0.0000
Groningen: one earthquake $M_L \geq 1.5$	2012	Repeat	0.3577	0.2753	0.4646	0.0000
Groningen: one earthquake $M_L \geq 1.5$	2013	Repeat	1.3186	0.9601	1.8111	0.0876
Groningen: one earthquake $M_L \geq 1.5$	2014	Repeat	0.9694	0.6494	1.4473	0.8794
Groningen: one earthquake $M_L \geq 1.5$	2015	Repeat	1.0017	0.6042	1.6606	0.9947

* Exposure = Number of Earthquakes $M_L \geq 1.5$
