# Supplementary material for: Immediate and long-term health impact of exposure to gas-mining induced earthquakes and related environmental stressors
Source: Eur J Public Health. 2021 Jan 26;31(4):715–21. doi: 10.1093/eurpub/ckaa244 (PMC8514061; doi:10.1093/eurpub/ckaa244)
Supplement: ckaa244_Supplementary_Data [file ckaa244_supplementary_data.zip › ckaa244-suppl_data/ejph-2020-04-om-0466-File007.pdf]

Supplementary File 3. Quarterly prevalence of health problems in the postal codes with $M_L \geq 3.0$ earthquakes (per 1000 patients)

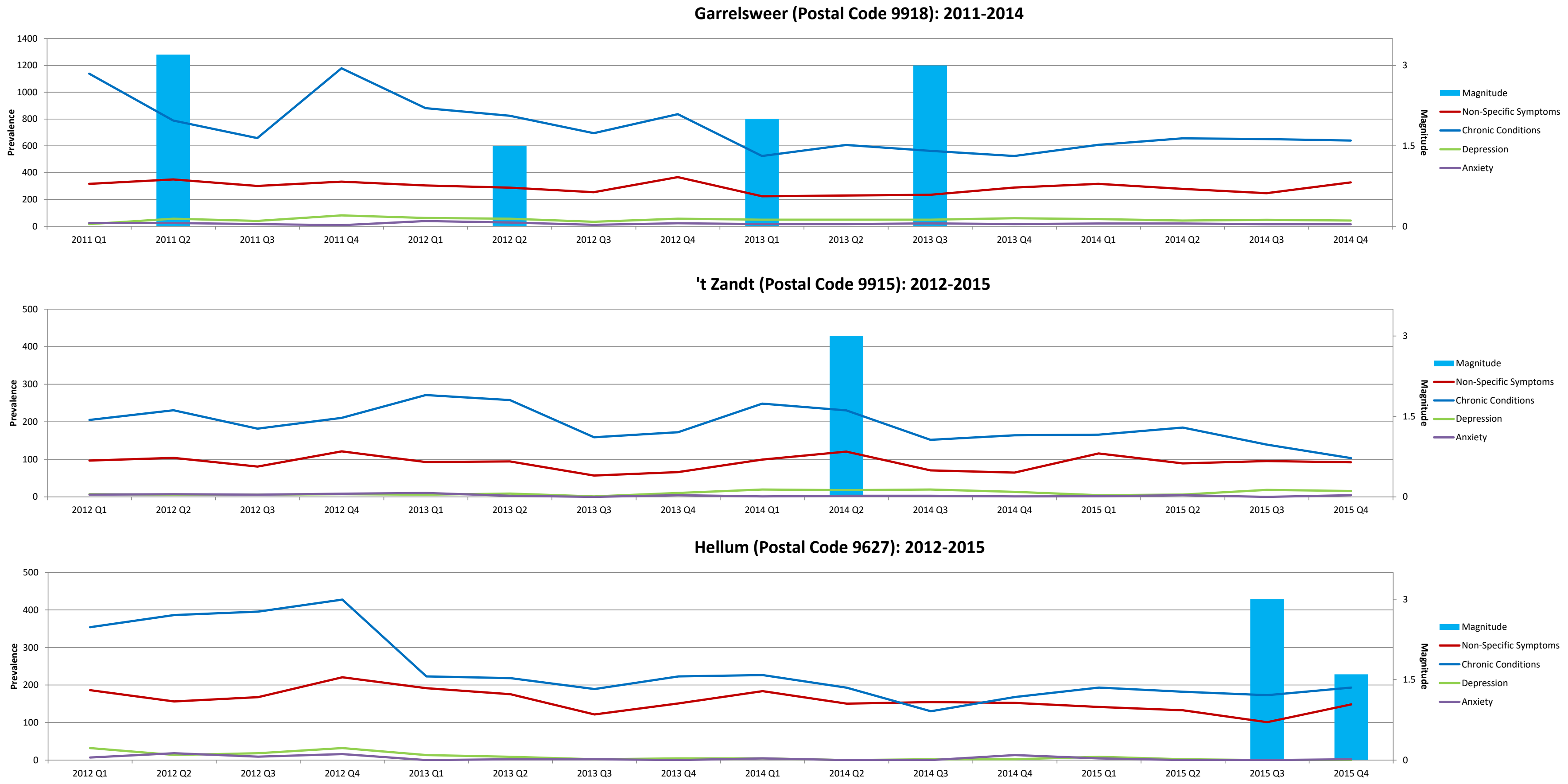

Note. The prevalence of non-specific symptoms, chronic conditions, anxiety and depression is displayed for postal codes with a $M_L \geq 3.0$ earthquake ($M_L \geq 1.5$ earthquakes are included in the figure if they occurred). In each postal code the prevalence decreases over time, whether the population was confronted with noticeable earthquakes or not. Again, the prevalence of stress reactions, social problems and suicidality recorded by general practitioners in these postal code areas was too low to include in the figure.
